# Supplementary material for: Spatial transcriptomics reveals discrete tumour microenvironments and autocrine loops within ovarian cancer subclones
Source: Nat Commun. 2024 Apr 3;15:2860. doi: 10.1038/s41467-024-47271-y (PMC10991508; doi:10.1038/s41467-024-47271-y)
Supplement: Supplementary file 1 — Supplementary Information [file 41467_2024_47271_MOESM1_ESM.pdf]

Supplementary Information for: Spatial transcriptomics reveals discrete tumour microenvironments and autocrine loops within ovarian cancer subclones

Authors: Elena Denisenko, Leanne de Kock, Adeline Tan, Aaron B. Beasley, Maria Beilin, Matthew E. Jones, Rui Hou, Dáithí Ó Muirí, Sanela Bilic, G. Raj K. A. Mohan, Stuart Salfinger, Simon Fox, Khaing P. W. Hmon, Yen Yeow, Youngmi Kim, Rhea John, Tami S. Gilderman, Emily Killingbeck, Elin S. Gray, Paul A. Cohen, Yu Yu, Alistair R. R. Forrest

This PDF file includes:

Supplementary Figures S1 to S24

Supplementary Notes 1 to 3.

Supplementary Fig. S1: Study design. Samples were collected during interval debulking surgery from patients who underwent 3-4 cycles of platinum and taxane treatment. Eight samples were profiled using 10x Genomics Visium and five samples using 10x Genomics 3' Gene Expression solution.

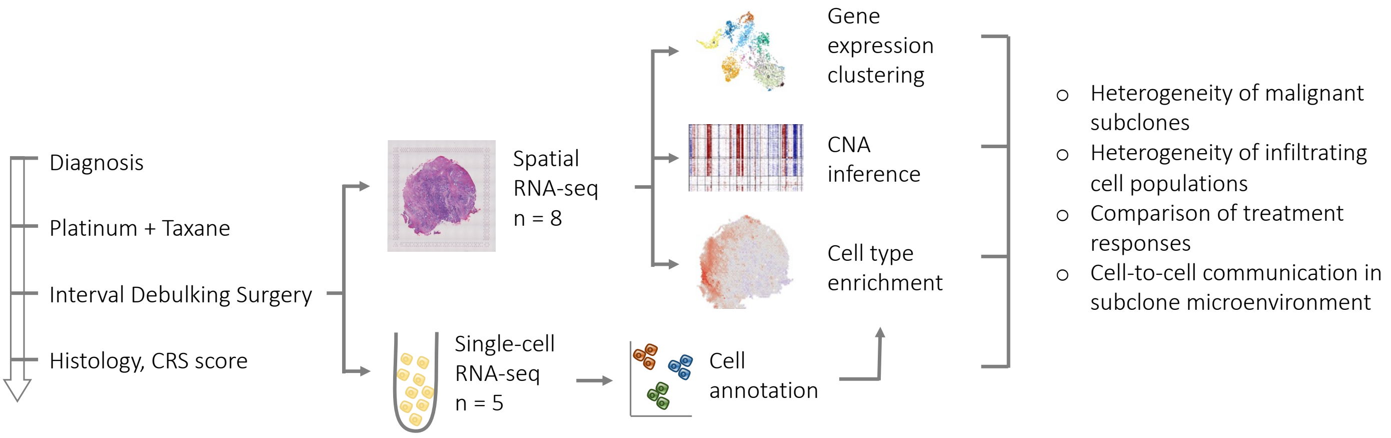

Supplementary Fig. S2: Correlation between RCTD cell type weights across all Visium spots and samples (n = 19,990). All but 3 correlation coefficients were significant with FDR < 0.05 (Benjamini-Hochberg correction) (insignificant: T cells and Fibro2, Fibro5 and Fibro2, Macrophages and Endothelial cells). Non-normalised cell type RCTD weights were used.

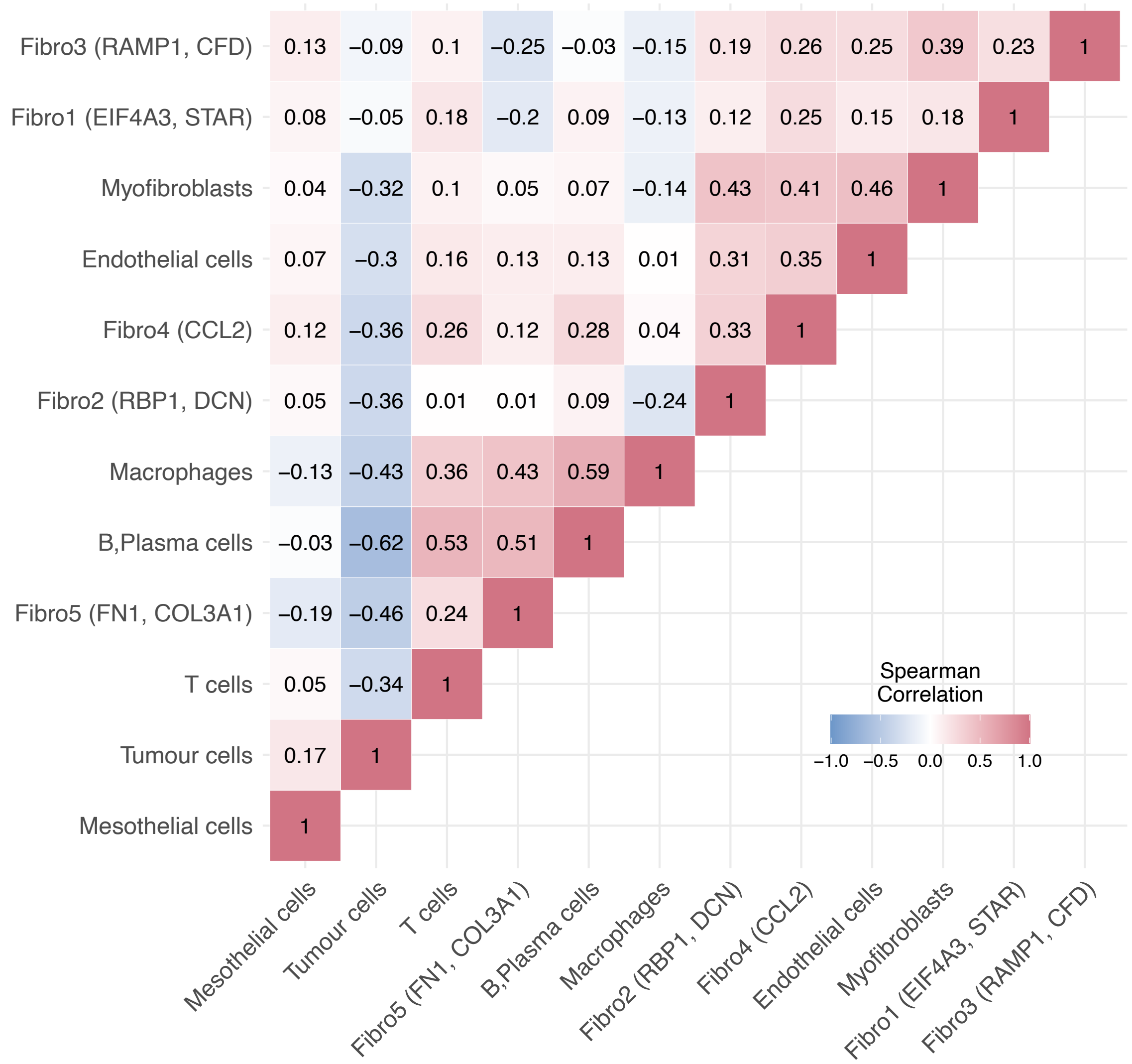

Supplementary Fig. S3: Spatial distribution of RCTD cell type weights in each of the Visium samples, using 12 cell populations from our scRNA-seq dataset as a reference.

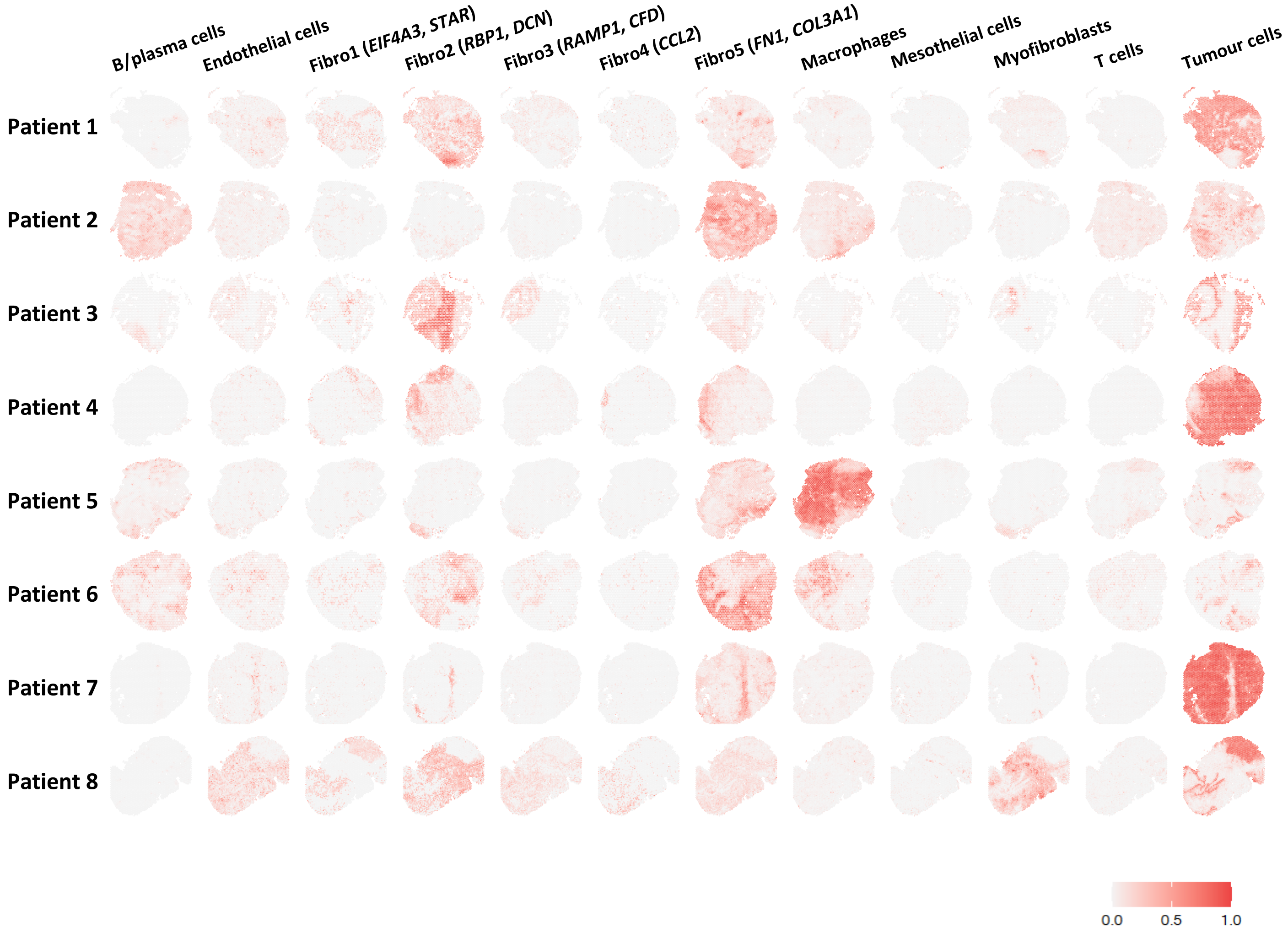

Supplementary Fig. S4: Spatial distribution of RCTD cell type weights in each of the Visium samples, using Zhang *et al.*[1] dataset as a reference. EOC: epithelial ovarian carcinoma. Average Spearman correlation with RCTD scores for scRNA-seq generated here: endothelial cells – 0.63, macrophages – 0.82, T cells – 0.52.

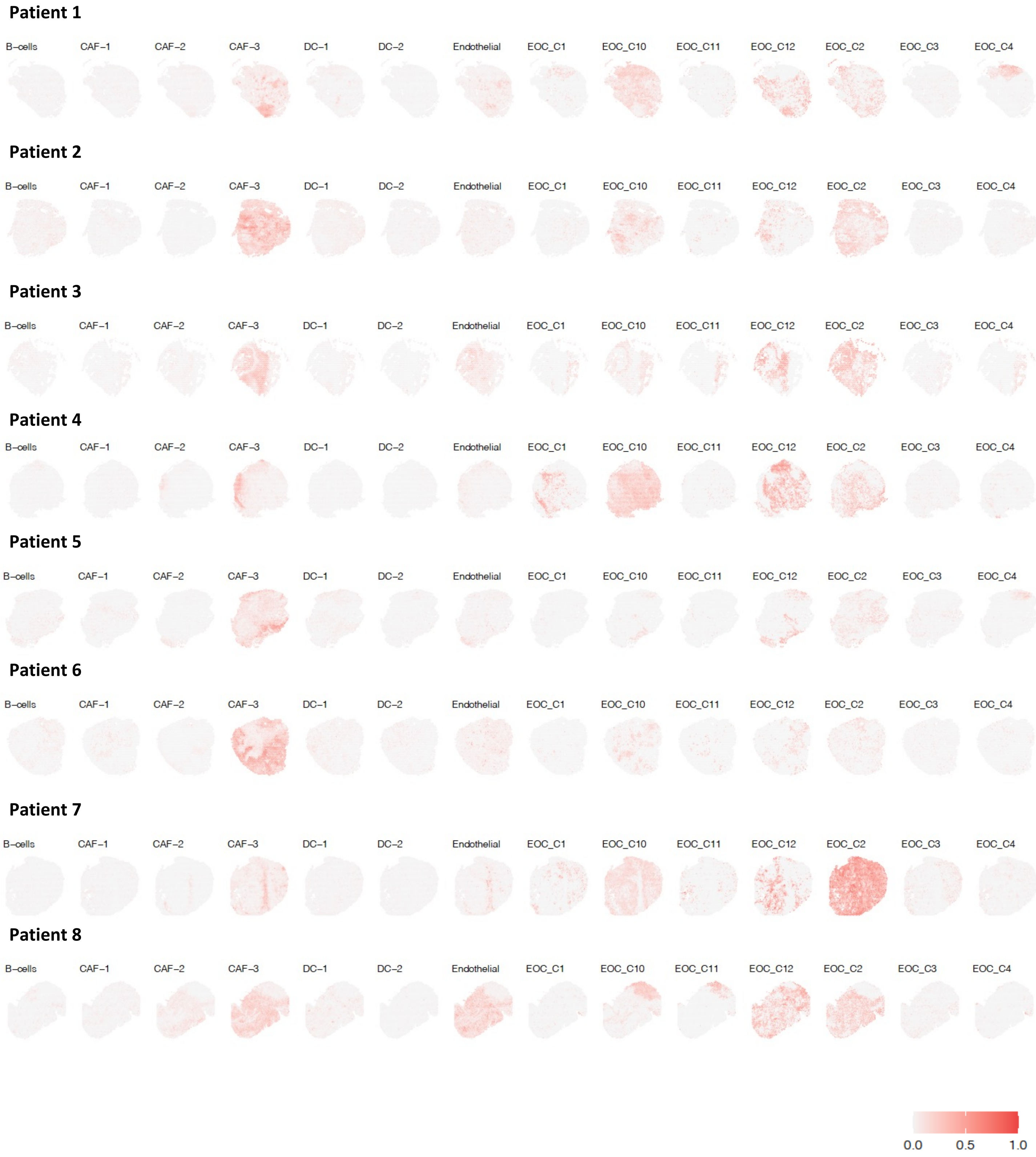

Supplementary Fig. S4: Spatial distribution of RCTD cell type weights in each of the Visium samples, using Zhang *et al.*[1] dataset as a reference. EOC: epithelial ovarian carcinoma. Average Spearman correlation with RCTD scores for scRNA-seq generated here: endothelial cells – 0.63, macrophages – 0.82, T cells – 0.52.

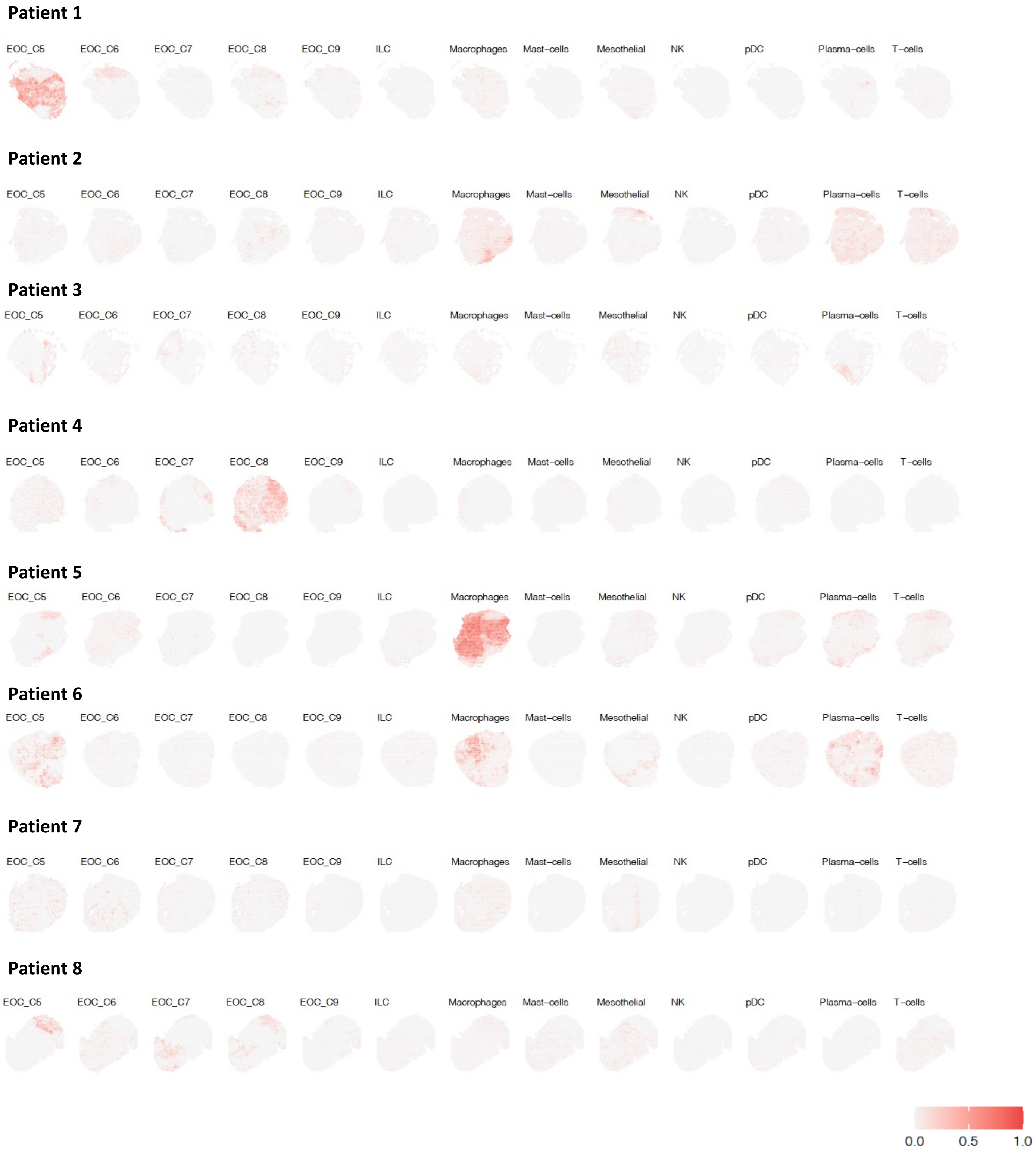

Supplementary Fig. S5: Patient 2 Visium sample. **a)** InferCNV heatmap showing inferred CNA profiles of Visium spots. Spot clusters are shown on the left. **b)** High-confidence CNAs predicted by inferCNV in clusters shown in (a). None were predicted for the cluster P2.2. **c)** Projection of the CNA-based clusters onto the tissue section as Visium spots. Blue spots correspond to a putative tumour subclone, grey spots are non-malignant regions with RCTD tumour scores <0.15, green and pink correspond to border regions. Scale bar = 1 mm. **d)** Distribution of Module scores for the four HGSOC molecular subtype signatures in each of the CNA-based clusters and the background spot set. Black lines are medians. Dotted lines show the value if all spots are combined as a pseudo-bulk. All pairwise comparisons between cluster module scores returned significant results, significance was determined using Mann-Whitney U test with Benjamini-Hochberg correction and 0.05 threshold (P2.1: n = 67, P2.2: n = 702, P2.3: n = 156, P2.background: n = 1007). **e)** Spatial distribution of Module scores for the four molecular subtype signatures. Scale bar = 1 mm.

In this patient we observe 3 clusters and predict one high-confidence CNA clonotype: P2.1 - Multiple high-confidence CNAs; P2.2 - No high-confidence CNAs; P2.3 - Likely border (all high-confidence CNAs are shared with P2.1).

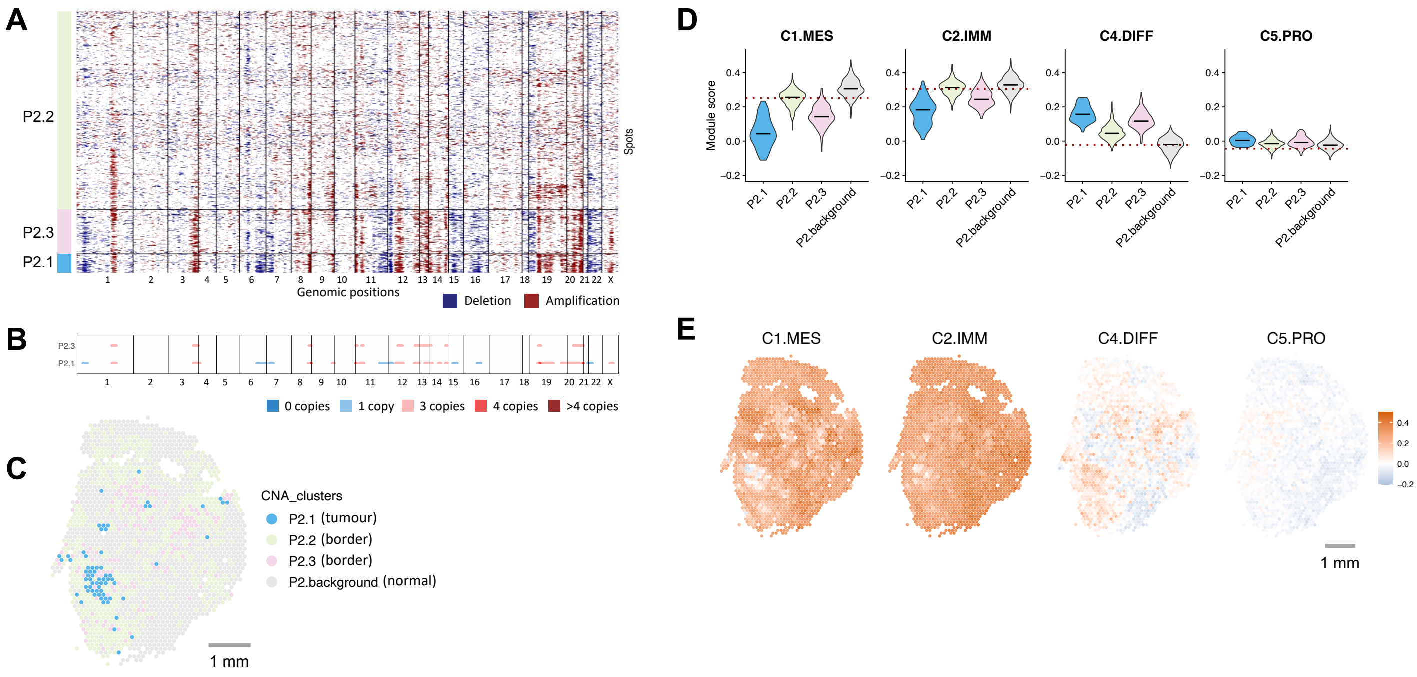

Supplementary Fig. S6: Patient 3 Visium sample. **a)** InferCNV heatmap showing inferred CNA profiles of Visium spots. Spot clusters are shown on the left. **b)** High-confidence CNAs predicted by inferCNV in clusters shown in (a). **c)** Projection of the CNA-based clusters onto the tissue section as Visium spots. Blue spots correspond to a putative tumour subclone, grey spots are non-malignant regions with RCTD tumour scores <0.15, green and pink correspond to border regions. Scale bar = 1 mm. **d)** Distribution of Module scores for the four HGSOC molecular subtype signatures in each of the CNA-based clusters and the background spot set. Black lines are medians. Dotted lines show the value if all spots are combined as a pseudo-bulk. ns indicates a pair of clusters where there was no significant difference in the module score, all other pairwise comparisons returned significant results; significance was determined using Mann-Whitney U test with Benjamini-Hochberg correction and 0.05 threshold (P3.1: n = 317, P3.2: n = 342, P3.3: n = 141, P3.background: n = 701). **e)** Spatial distribution of Module scores for the four molecular subtype signatures. Scale bar = 1 mm.

In this patient we observe 3 clusters and predict one high-confidence CNA clonotype: P3.1 - Multiple high-confidence CNAs, multiple CNAs not observed in P3.3; P3.2 - Likely border (one small high-confidence deletion on Chr 14); P3.3 - Likely border (one small high-confidence amplification on Chr 14, all other high-confidence CNAs are seen in P3.1).

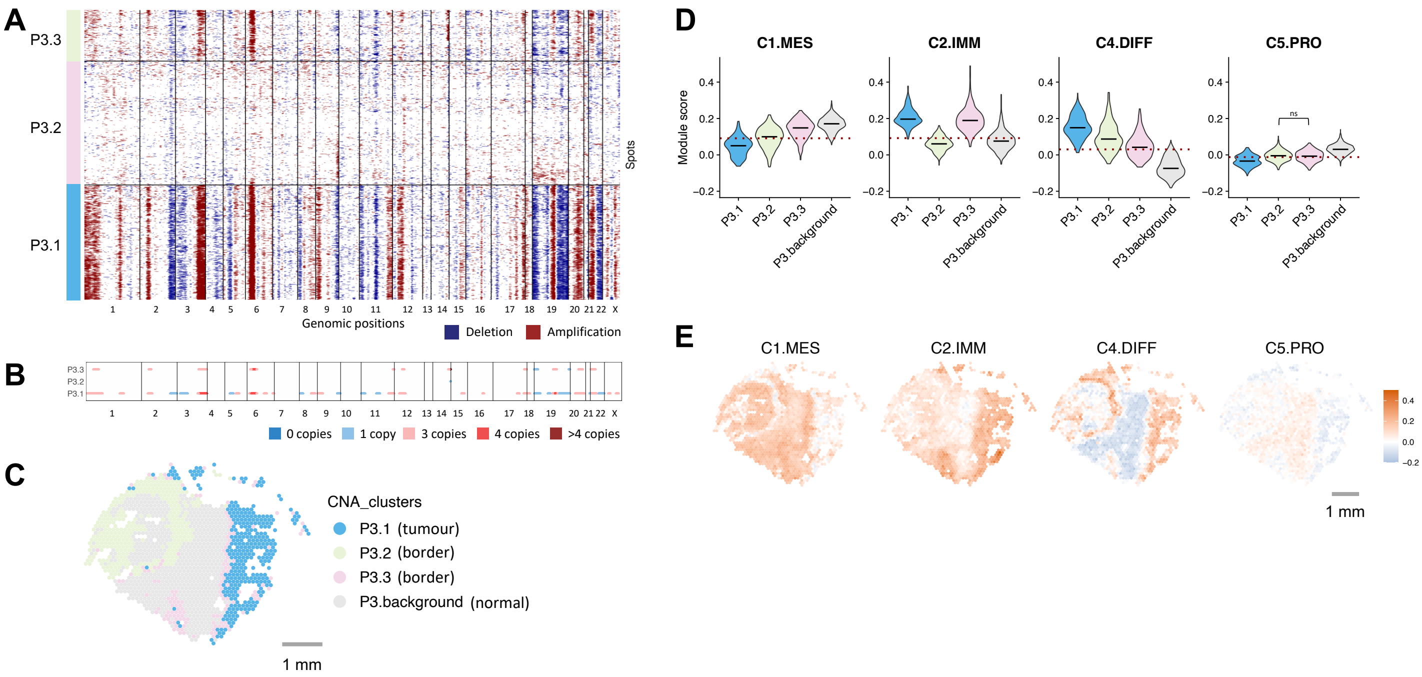

Supplementary Fig. S7: Patient 4 Visium sample. **a)** InferCNV heatmap showing inferred CNA profiles of Visium spots. Spot clusters are shown on the left. **b)** High-confidence CNAs predicted by inferCNV in clusters shown in (a). **c)** Projection of the CNA-based clusters onto the tissue section as Visium spots. Blue and red spots correspond to putative tumour subclones, grey spots are non-malignant regions with RCTD tumour scores <0.15, green and pink correspond to border regions. Rectangles indicate areas shown in (f). Scale bar = 1 mm. **d)** Distribution of Module scores for the four HGSOC molecular subtype signatures in each of the CNA-based clusters and the background spot set. Black lines are medians. Dotted lines show the value if all spots are combined as a pseudo-bulk. ns indicates 3 pairs of clusters where there was no significant difference in the module score, all other pairwise comparisons returned significant results; significance was determined using Mann-Whitney U test with Benjamini-Hochberg correction and 0.05 threshold (P4.1: n = 2066, P4.2: n = 357, P4.3: n = 313, P4.4: n = 160, P4.background: n = 104). **e)** Spatial distribution of Module scores for the four molecular subtype signatures. Scale bar = 1 mm. **f)** Representative tissue areas for two subclones. Hematoxylin (blue) and eosin (red) staining (HnE) and histopathological expert annotation using QuPath are shown, red corresponds to malignant cells, green corresponds to stroma. Location of these areas on the tissue is shown by rectangles in (c). Scale bar = 0.1 mm.

In this patient we observe 4 clusters and predict two high-confidence CNA clonotypes: P4.1 - Multiple high confidence CNAs including a unique amplification on Chr X; P4.2 - Multiple high confidence CNAs including a unique CNA on Chr 14 and stronger amplification on Chr 1; P4.3 - Likely border (three high-confidence CNAs shared with P4.1 and P4.2, one unique high-confidence CNA predicted on Chr 8 but seen in P4.1 (but not called as significant); P4.4 - Likely border (shares Chr X amplification from P4.1).

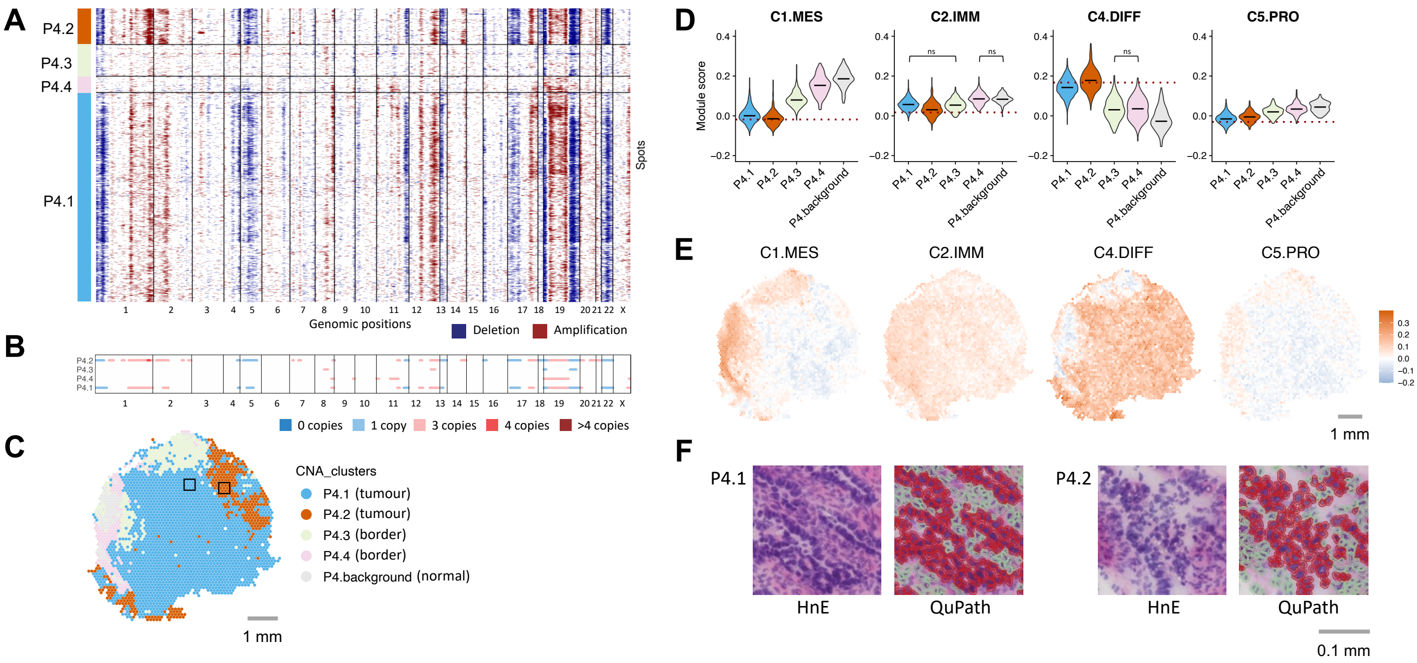

Supplementary Fig. S8: Patient 5 Visium sample. **a)** InferCNV heatmap showing inferred CNA profiles of Visium spots. Spot clusters are shown on the left. **b)** High-confidence CNAs predicted by inferCNV in clusters shown in (a). **c)** Projection of the CNA-based clusters onto the tissue section as Visium spots. Blue and red spots correspond to putative tumour subclones, grey spots are non-malignant regions with RCTD tumour scores <0.15, green corresponds to border regions. Rectangles indicate areas shown in (d). Scale bar = 1 mm. **d)** Representative tissue areas for two subclones. Hematoxylin (blue) and eosin (red) staining (HnE) and histopathological expert annotation using QuPath are shown, red corresponds to malignant cells, green corresponds to stroma. Location of these areas on the tissue is shown by rectangles in (c). Scale bar = 0.1 mm.

In this patient we observe 3 clusters and predict two high-confidence CNA clonotypes: P5.1 - Unique high-confidence CNAs on Chr 11, 12; P5.2 - Multiple unique high-confidence CNAs (e.g., on Chr 1, 2, 3, 4, 5, 7, 17), P5.3 - Likely border (one small high-confidence deletion on Chr 20 seen in P5.1 and P5.2).

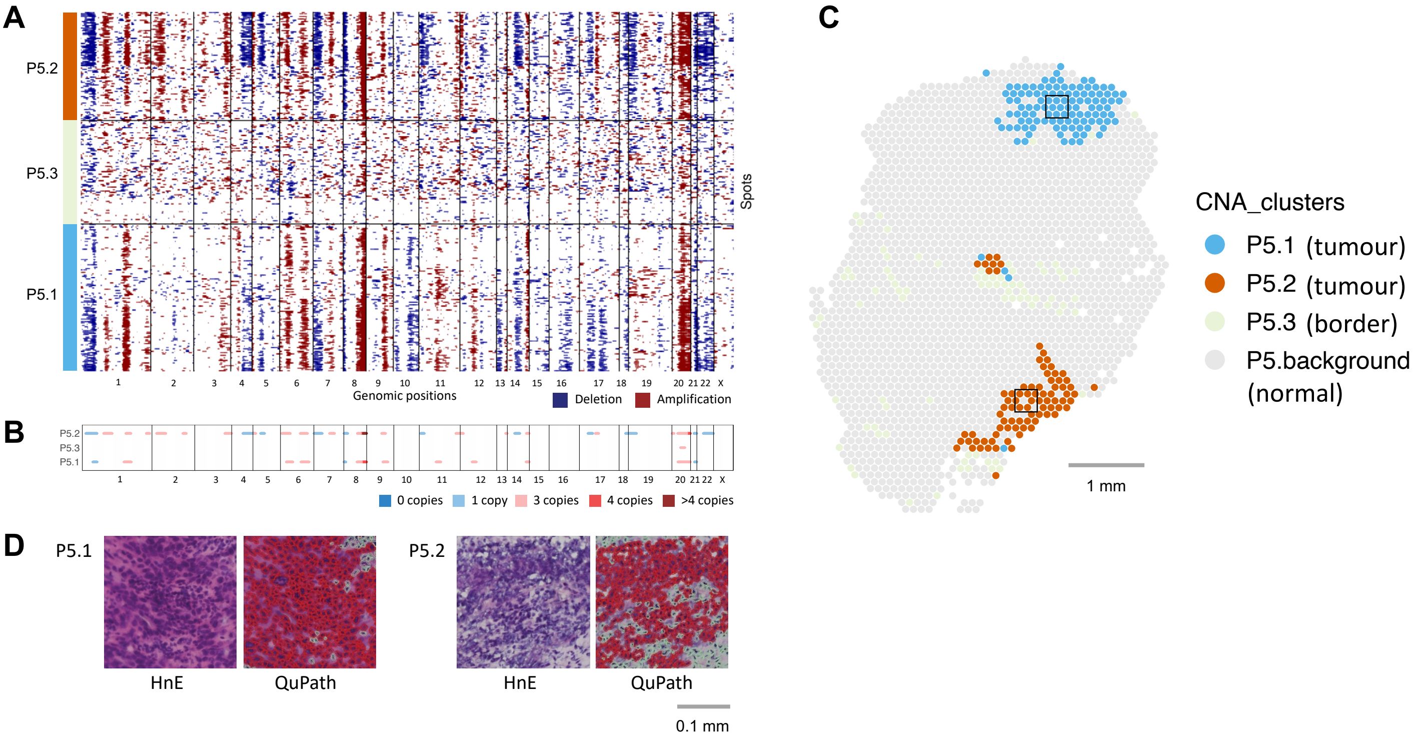

Supplementary Fig. S9: Patient 6 Visium sample. **a)** InferCNV heatmap showing inferred CNA profiles of Visium spots. Spot clusters are shown on the left. **b)** High-confidence CNAs predicted by inferCNV in clusters shown in (a). **c)** Projection of the CNA-based clusters onto the tissue section as Visium spots. Blue and red spots correspond to putative tumour subclones, grey spots are non-malignant regions with RCTD tumour scores <0.15, green and pink correspond to border regions. Rectangles indicate areas shown in (f). Scale bar = 1 mm. **d)** Distribution of Module scores for the four HGSOC molecular subtype signatures in each of the CNA-based clusters and the background spot set. Black lines are medians. Dotted lines show the value if all spots are combined as a pseudo-bulk. ns indicates 5 pairs of clusters where there was no significant difference in the module score, all other pairwise comparisons returned significant results; significance was determined using Mann-Whitney U test with Benjamini-Hochberg correction and 0.05 threshold (P6.1: n = 58, P6.2: n = 31, P6.3: n = 89, P6.4: n = 65, P6.background: n = 1548). **e)** Spatial distribution of Module scores for the four molecular subtype signatures. Scale bar = 1 mm. **f)** Representative tissue areas for two subclones. Hematoxylin (blue) and eosin (red) staining (HnE) and histopathological expert annotation using QuPath are shown, red corresponds to malignant cells, green corresponds to stroma. Location of these areas on the tissue is shown by rectangles in (c). Scale bar = 0.1 mm.

In this patient we observe 4 clusters and predict two high-confidence CNA clonotypes: P6.1 - Multiple unique high-confidence CNAs (e.g., on Chr 8, 11, 14, 16, 17, X); P6.2 - Multiple unique high-confidence CNAs (e.g. on Chr 1, 2, 7); P6.3 - Likely border containing clonotype from P6.2 (no unique high-confidence CNAs); P6.4 - Likely border containing clonotype from P6.1 (no unique high-confidence CNAs).

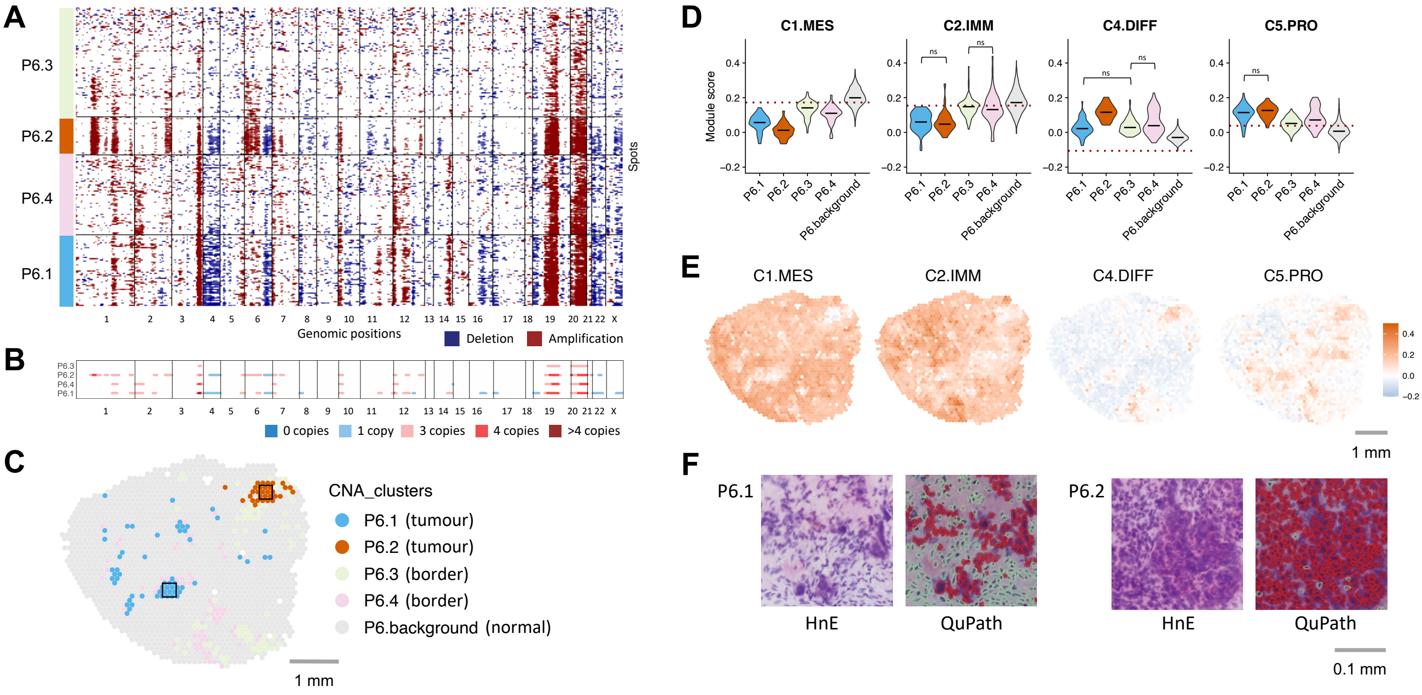

Supplementary Fig. S10: Patient 7 Visium sample. **a)** InferCNV heatmap showing inferred CNA profiles of Visium spots. Spot clusters are shown on the left. **b)** High-confidence CNAs predicted by inferCNV in clusters shown in (a). None were predicted for the cluster P7.3. **c)** Projection of the CNA-based clusters onto the tissue section as Visium spots. Blue and red spots correspond to putative tumour subclones, grey spots are non-malignant regions with RCTD tumour scores <0.15, green corresponds to border regions. Rectangles indicate areas shown in (f). Scale bar = 1 mm. **d)** Distribution of Module scores for the four HGSOC molecular subtype signatures in each of the CNA-based clusters and the background spot set. Black lines are medians. Dotted lines show the value if all spots are combined as a pseudo-bulk. All pairwise comparisons between cluster module scores returned significant results, significance was determined using Mann-Whitney U test with Benjamini-Hochberg correction and 0.05 threshold (P7.1: n = 3083, P7.2: n = 274, P7.3: n = 163, P7.background: n = 64). **e)** Spatial distribution of Module scores for the four molecular subtype signatures. Scale bar = 1 mm. **f)** Representative tissue areas for two subclones. Hematoxylin (blue) and eosin (red) staining (HnE) and histopathological expert annotation using QuPath are shown, red corresponds to malignant cells, green corresponds to stroma. Location of these areas on the tissue is shown by rectangles in (c). Scale bar = 0.1 mm.

In this patient we observe 3 clusters and predict two high-confidence CNA clonotypes: P7.1 - Multiple unique high-confidence CNAs (e.g., on Chr 5, 6, 13, 14, 18); P7.2 - Multiple unique high-confidence CNAs (e.g., on Chr 4, 11, 19); P7.3 - No high-confidence CNAs.

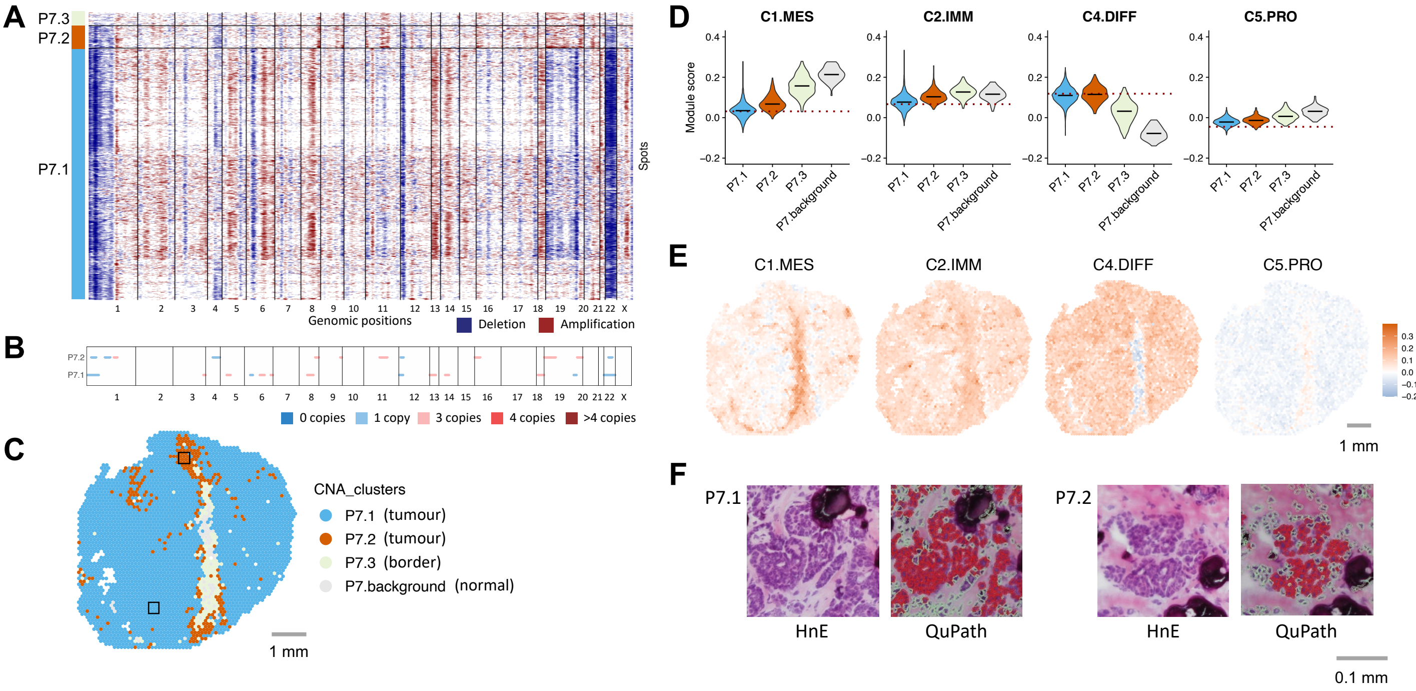

Supplementary Fig. S11: Patient 8 Visium sample. **a)** InferCNV heatmap showing inferred CNA profiles of Visium spots. Spot clusters are shown on the left. **b)** High-confidence CNAs predicted by inferCNV in clusters shown in (a). None were predicted for the cluster P8.2. **c)** Projection of the CNA-based clusters onto the tissue section as Visium spots. Blue spots correspond to a putative tumour subclone, grey spots are non-malignant regions with RCTD tumour scores <0.15, green corresponds to border regions. Scale bar = 1 mm. **d)** Distribution of Module scores for the four HGSOC molecular subtype signatures in each of the CNA-based clusters and the background spot set. Black lines are medians. Dotted lines show the value if all spots are combined as a pseudo-bulk. ns indicates a pair of clusters where there was no significant difference in the module score, all other pairwise comparisons returned significant results; significance was determined using Mann-Whitney U test with Benjamini-Hochberg correction and 0.05 threshold (P8.1: n = 476, P8.2: n = 814, P8.background: n = 1878). **e)** Spatial distribution of Module scores for the four molecular subtype signatures. Scale bar = 1 mm.

In this patient we observe 2 clusters and predict one high-confidence CNA clonotype: P8.1 - Multiple high-confidence CNAs, P8.2 - No high-confidence CNAs.

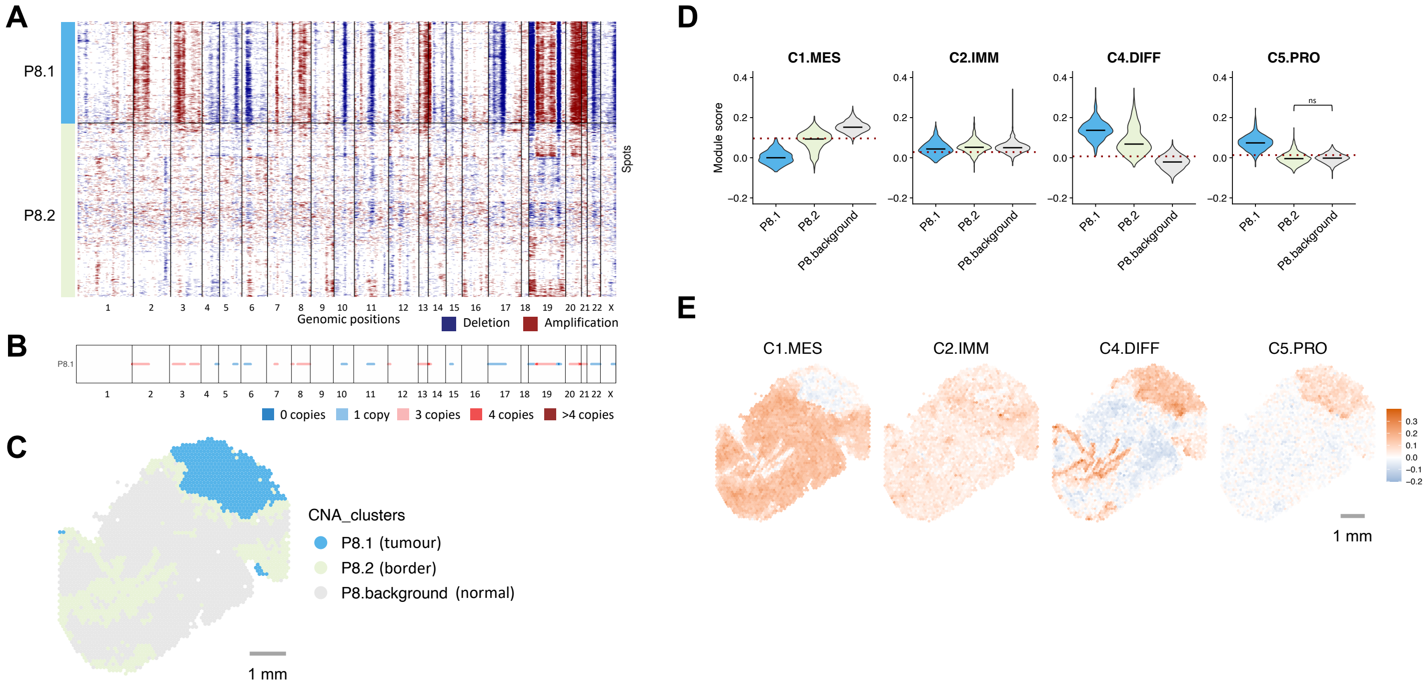

Supplementary Fig. S12: High-confidence CNAs predicted by inferCNV in eight Visium samples in CNA-based clusters corresponding to putative malignant subclones. Red corresponds to amplification, blue to deletion. Three top rows show recurrent amplifications (red) and deletions (blue) identified at three frequencies in the 579 HGSOC tumour genomes sequenced by the TCGA (data was accessed from cBioPortal[2]). Note, TCGA CNAs are shown only for genes tested for CNA by inferCNV in the Visium dataset.

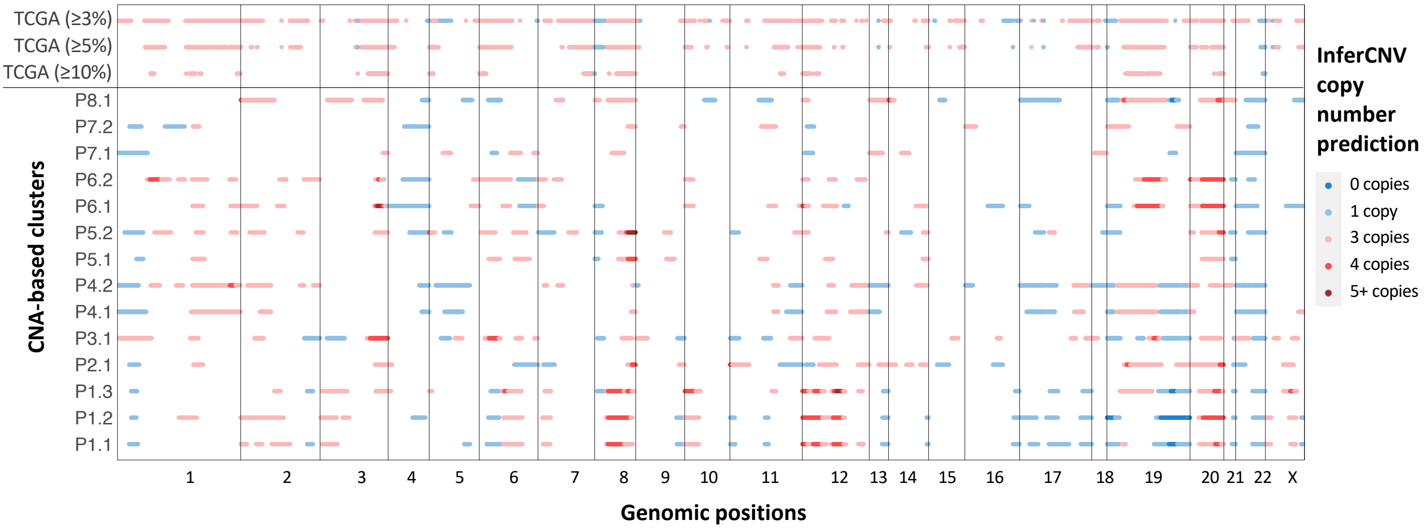

Supplementary Fig. S13: IchorCNA profiles for microdissected tissue areas corresponding to three tumour subclones (P1.1, P1.2, P1.3) and non-malignant tissue (P1.background). a) Tissue section showing extracted tissue fragments. Scale bar = 1 mm. **b)** IchorCNA profiles.

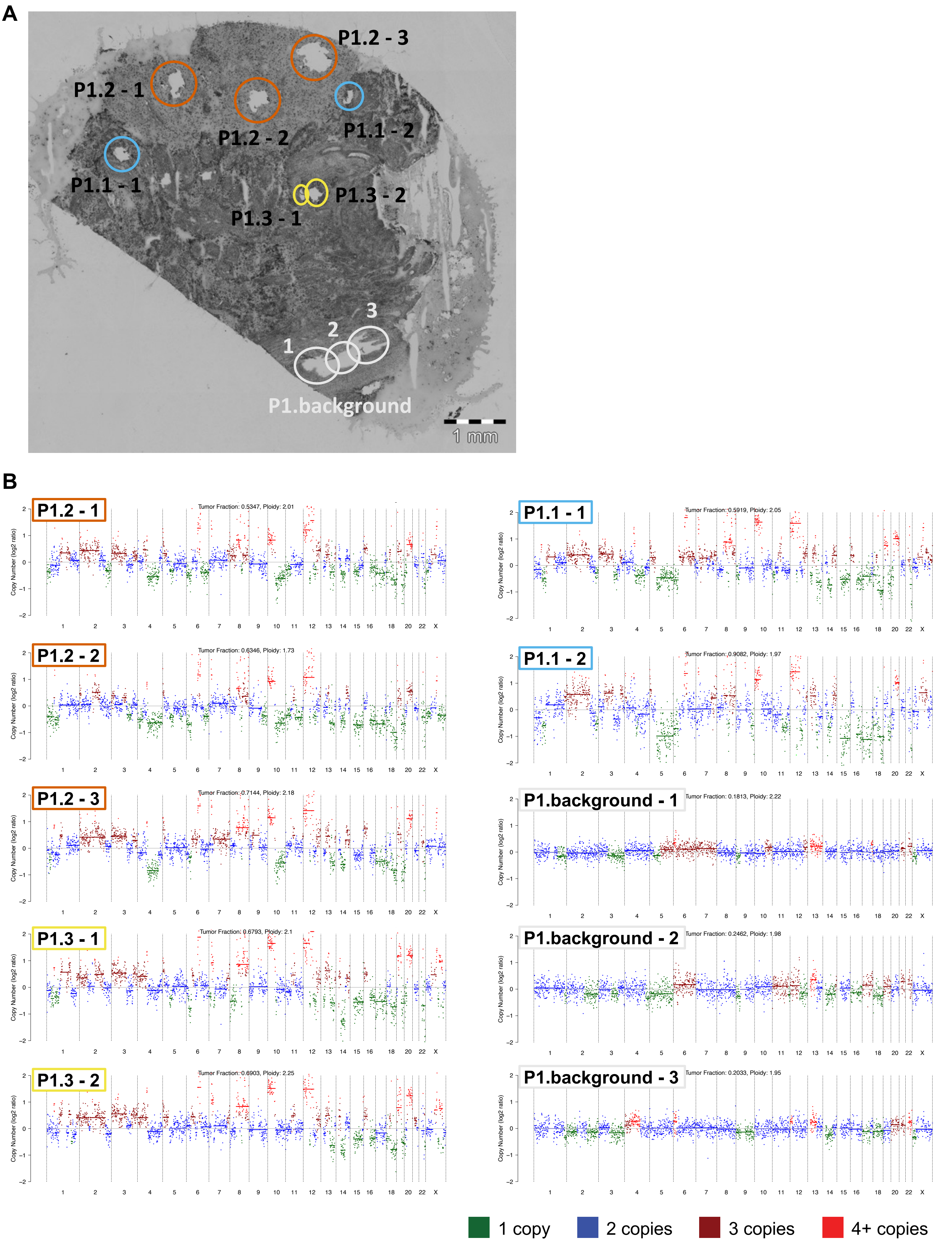

Supplementary Fig. S14: Infiltrating cell population differences across malignant CNA-based clusters. Adjusted RCTD cell type weights are shown (normalised to sum up to 1 for non-tumour cell types), averaged across spots for each malignant cluster. Black lines indicate cell types with significantly different infiltration patterns between clusters of the same patient. Statistical significance was determined by permutation testing, Benjamini-Hochberg FDR correction procedure was applied and the threshold was set to 0.05 (P1.1: n = 590, P1.2: n = 371, P1.3: n = 51, P4.1: n = 2066, P4.2: n = 357 , P5.1: n = 114, P5.2: n = 84 , P6.1: n = 58, P6.2: n = 31, P7.1: n = 3083, P7.2: n = 274).

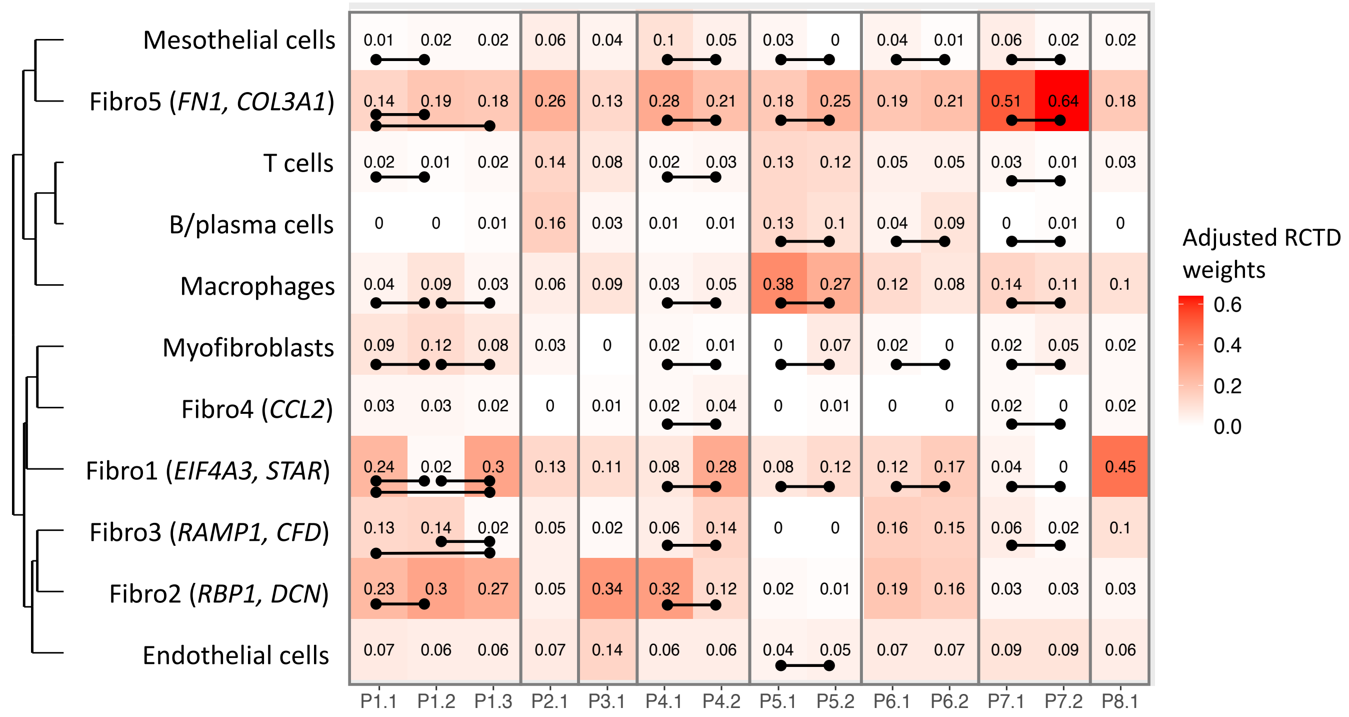

Supplementary Fig. S15: Overview of the CosMx dataset. a) Immunofluorescence composite images for 20 Fields Of View. Green – PanCK, Red – CD45, Yellow – CD3, Blue – DAPI. **b)** Number of cell segments identified by Cellpose in each Field Of View (FOV) after/before filtering (≥100 transcripts). Scale bar = 1 mm.

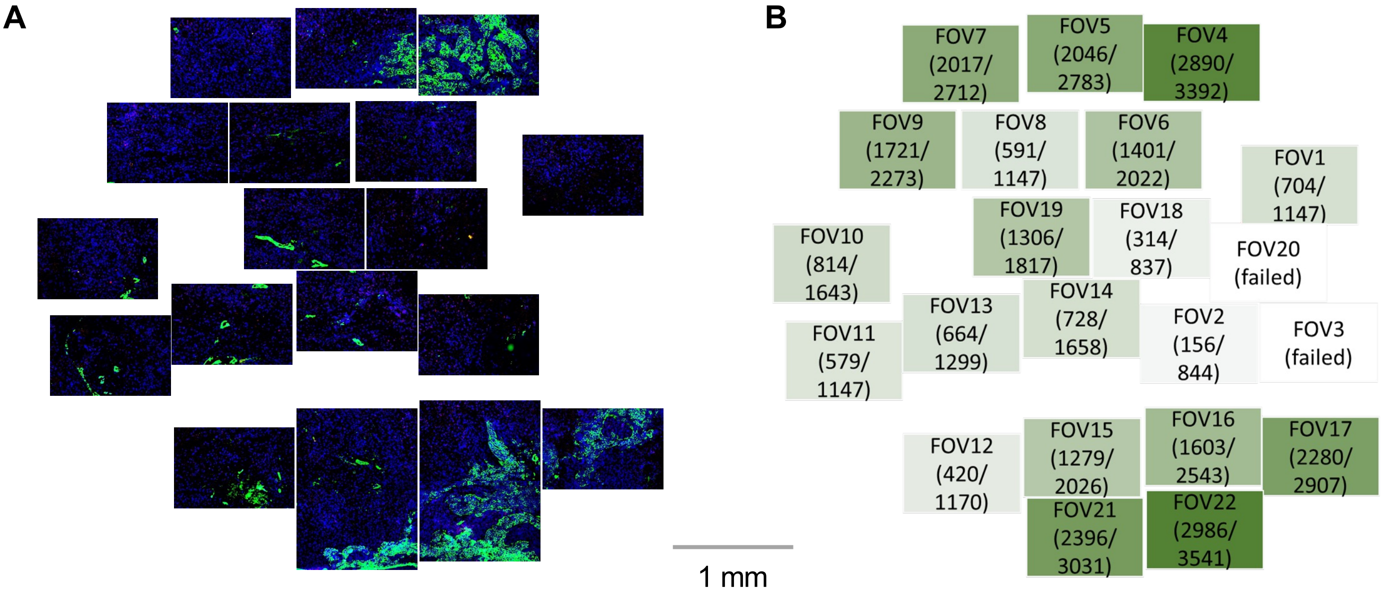

Supplementary Fig. S16: InferCNV heatmaps showing inferred CNA profiles for previously published HGSOC Visium data. a-b) Stur *et al.*[3] study. Samples from six excellent (ER) and six poor responders (PR) to neoadjuvant chemotherapy are shown. As a reference for inferCNV, we used a combined set of 275 spots from all 12 samples, for which RCTD tumour cell weights were less or equal to 0.15. **c)** Ferri-Borgogno *et al.*[4] study. As a reference for inferCNV, we used a combined set of 441 spots from all 4 samples, for which RCTD tumour cell weights were less or equal to 0.15. Note, subclones are predicted in dA10 and dA12 samples from short term survivors and high-confidence CNAs are shown below the heatmaps.

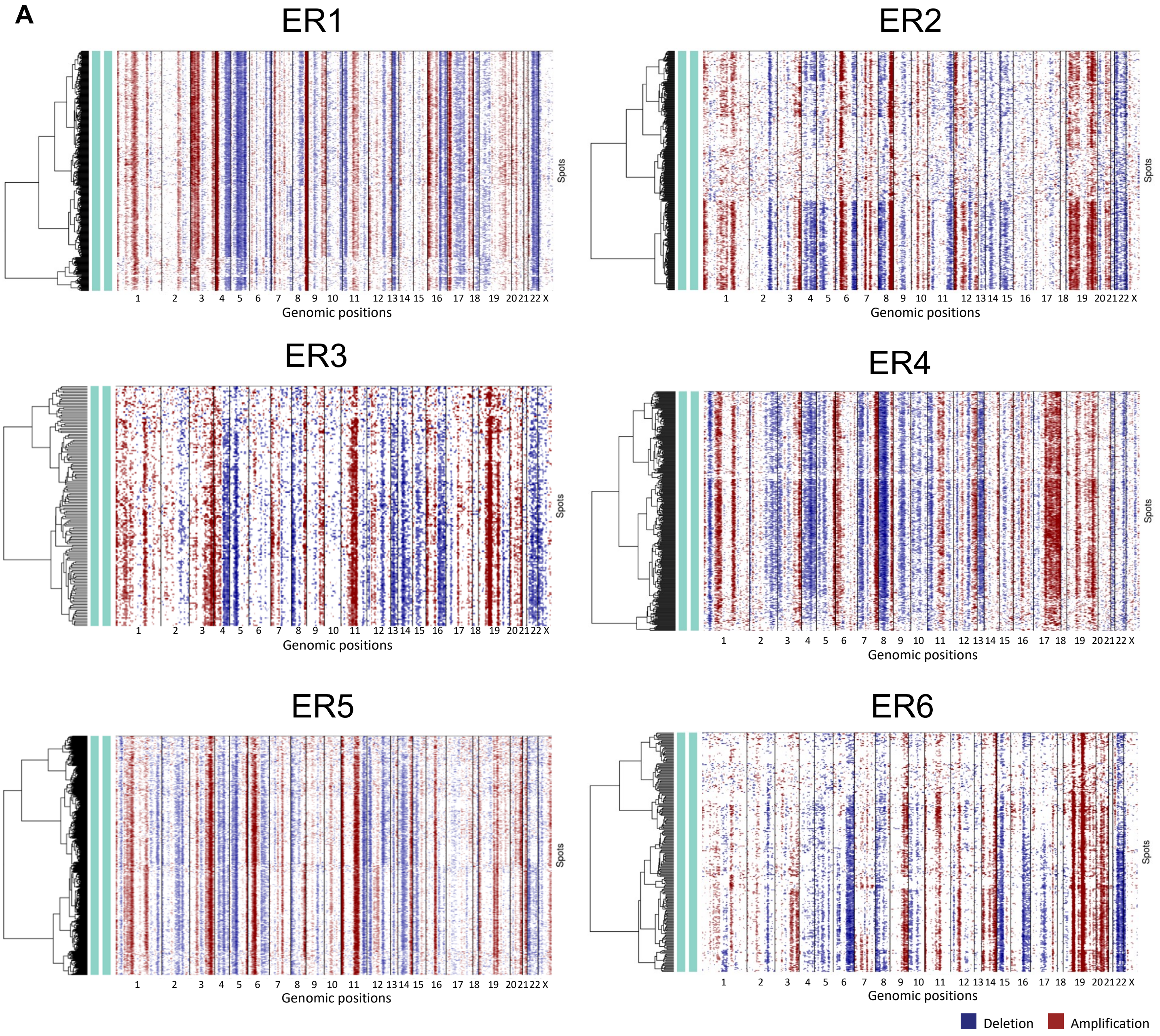

Supplementary Fig. S16: InferCNV heatmaps showing inferred CNA profiles for previously published HGSOC Visium data. a-b) Stur *et al.*[3] study. Samples from six excellent (ER) and six poor responders (PR) to neoadjuvant chemotherapy are shown. As a reference for inferCNV, we used a combined set of 275 spots from all 12 samples, for which RCTD tumour cell weights were less or equal to 0.15. **c)** Ferri-Borgogno *et al.*[4] study. As a reference for inferCNV, we used a combined set of 441 spots from all 4 samples, for which RCTD tumour cell weights were less or equal to 0.15. Note, subclones are predicted in dA10 and dA12 samples from short term survivors and high-confidence CNAs are shown below the heatmaps.

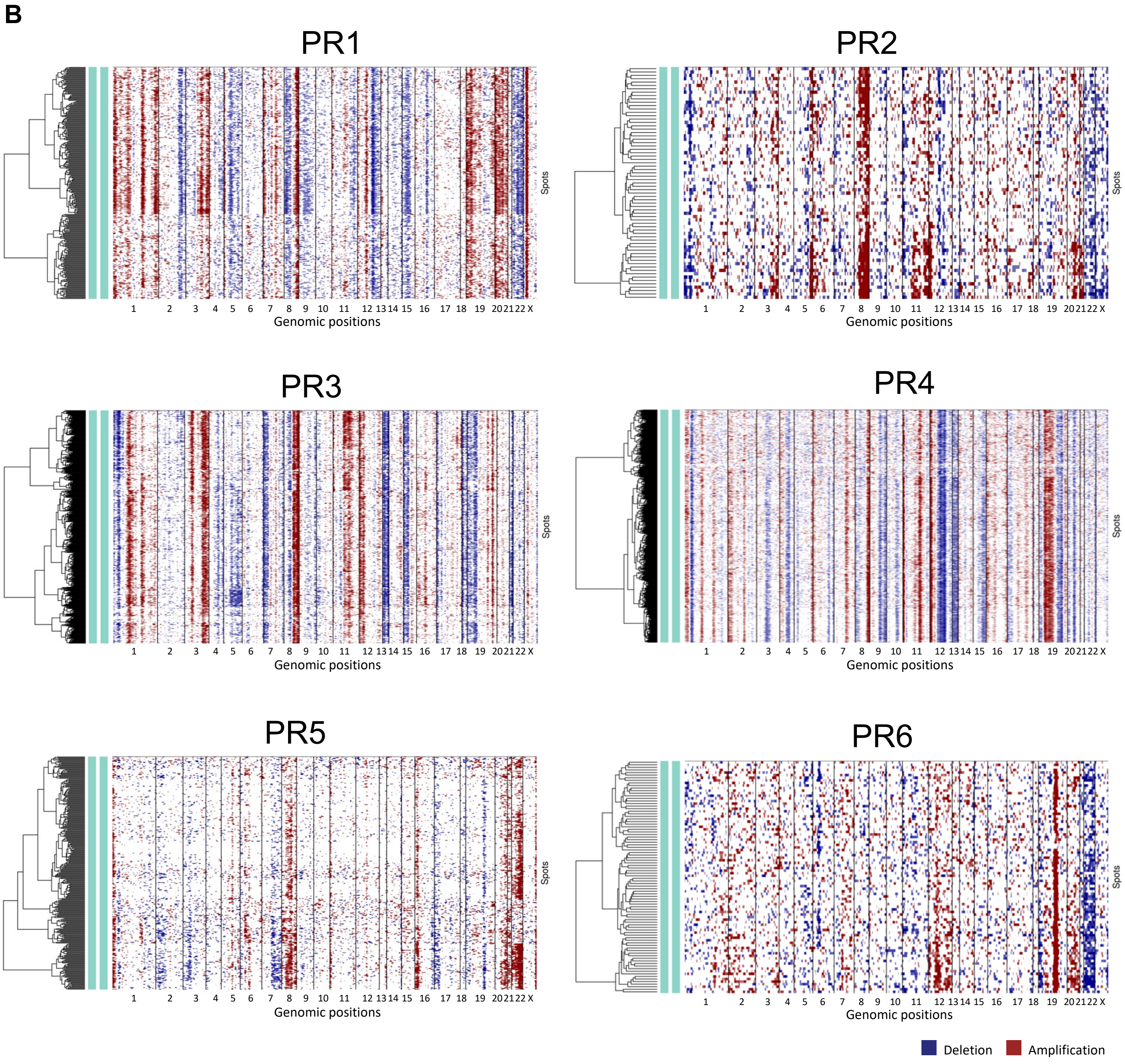

Supplementary Fig. S16: InferCNV heatmaps showing inferred CNA profiles for previously published HGSOC Visium data. a-b) Stur *et al.*[3] study. Samples from six excellent (ER) and six poor responders (PR) to neoadjuvant chemotherapy are shown. As a reference for inferCNV, we used a combined set of 275 spots from all 12 samples, for which RCTD tumour cell weights were less or equal to 0.15. **c)** Ferri-Borgogno *et al.*[4] study. As a reference for inferCNV, we used a combined set of 441 spots from all 4 samples, for which RCTD tumour cell weights were less or equal to 0.15. Note, subclones are predicted in dA10 and dA12 samples from short term survivors and high-confidence CNAs are shown below the heatmaps.

c

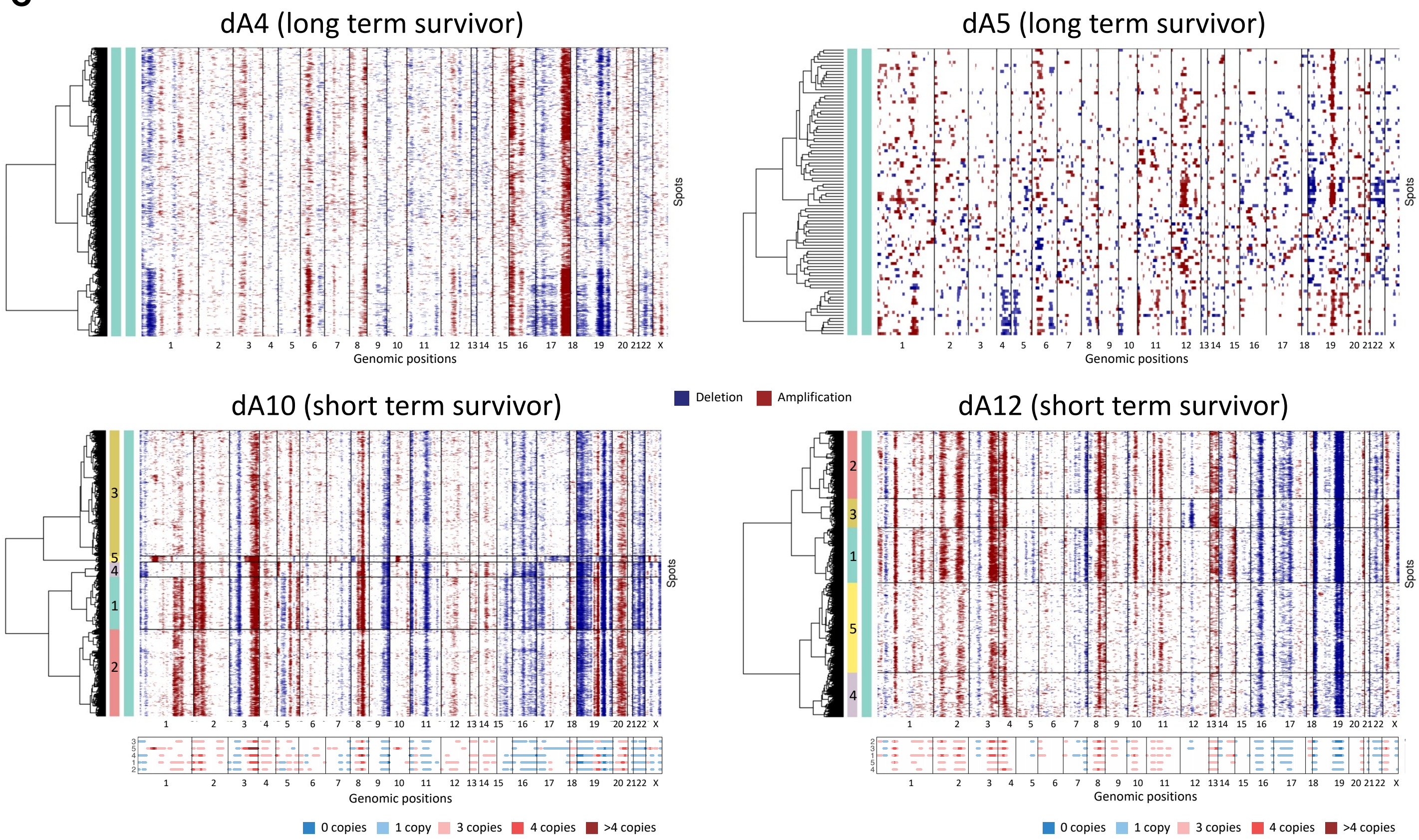

Supplementary Fig. S17: Annotation of the single-cell RNA-seq HGSOC dataset (n = 5 samples, 17,192 cells). a) UMAP showing top-level cell type annotations inferred by scMatch with Olbrecht *et al.*[5] reference dataset. **b)** Differentially expressed genes (DEGs) identified in the top-level cell types; top DEGs based on the ratio of detection rates are shown for each cell type. **c)** UMAP showing subclustering of fibroblasts and stromal cells. **d)** DEGs identified in the subclusters shown in (**c**); top DEGs based on the ratio of detection rates are shown for each cell type.

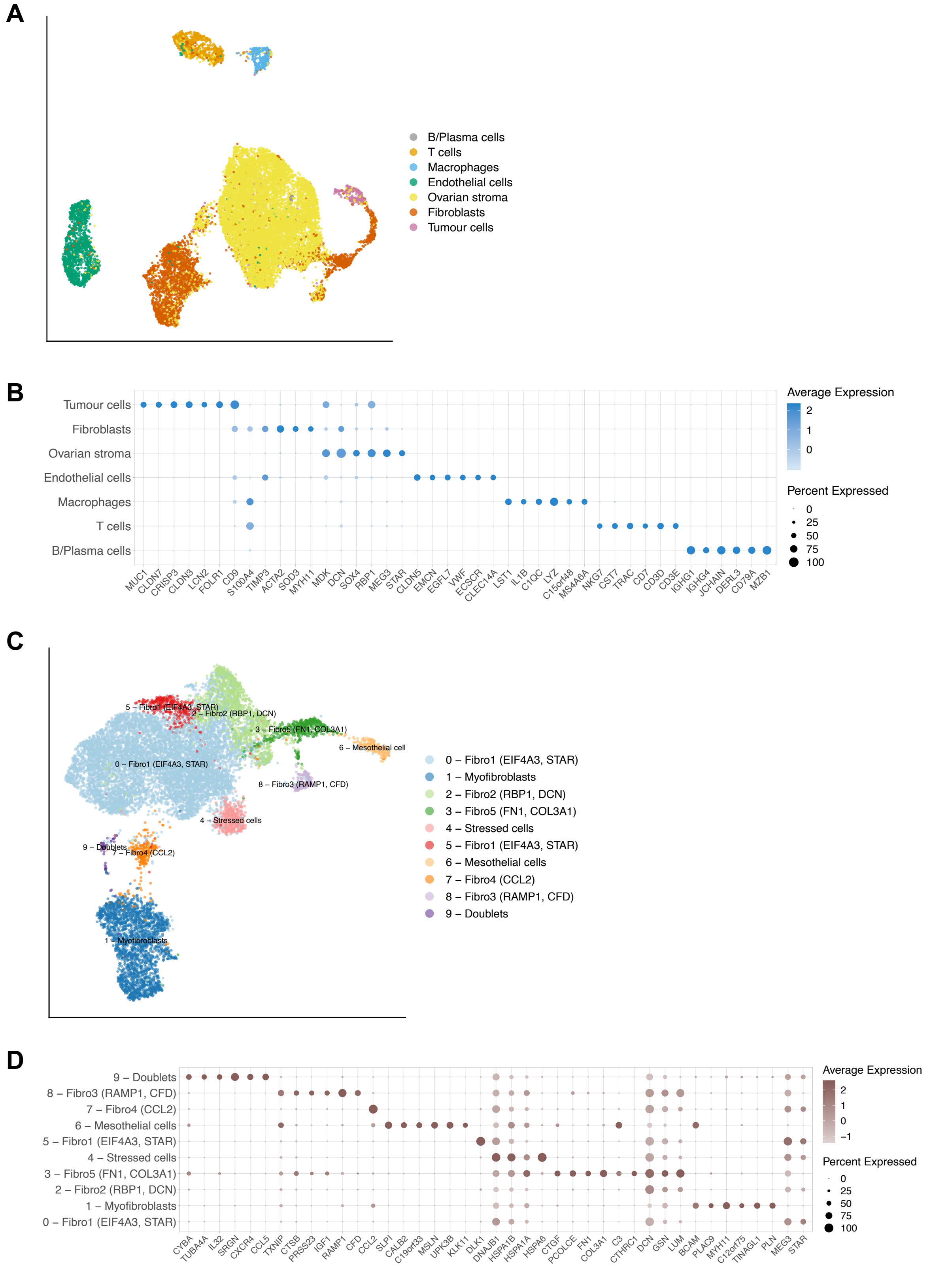

Supplementary Fig. S18: Fine-grain annotations of the scRNA-seq dataset (n = 5 samples, 17,192 cells). a) UMAP showing 12 cell types. **b)** Top differentially expressed genes (DEGs) over-expressed in each of the cell types, ranked based on log_eFC. **c)** Top DEGs over-expressed in each of the cell types, ranked based on the ratio of detection rates. DEGs were calculated for each cell type vs all other cells.

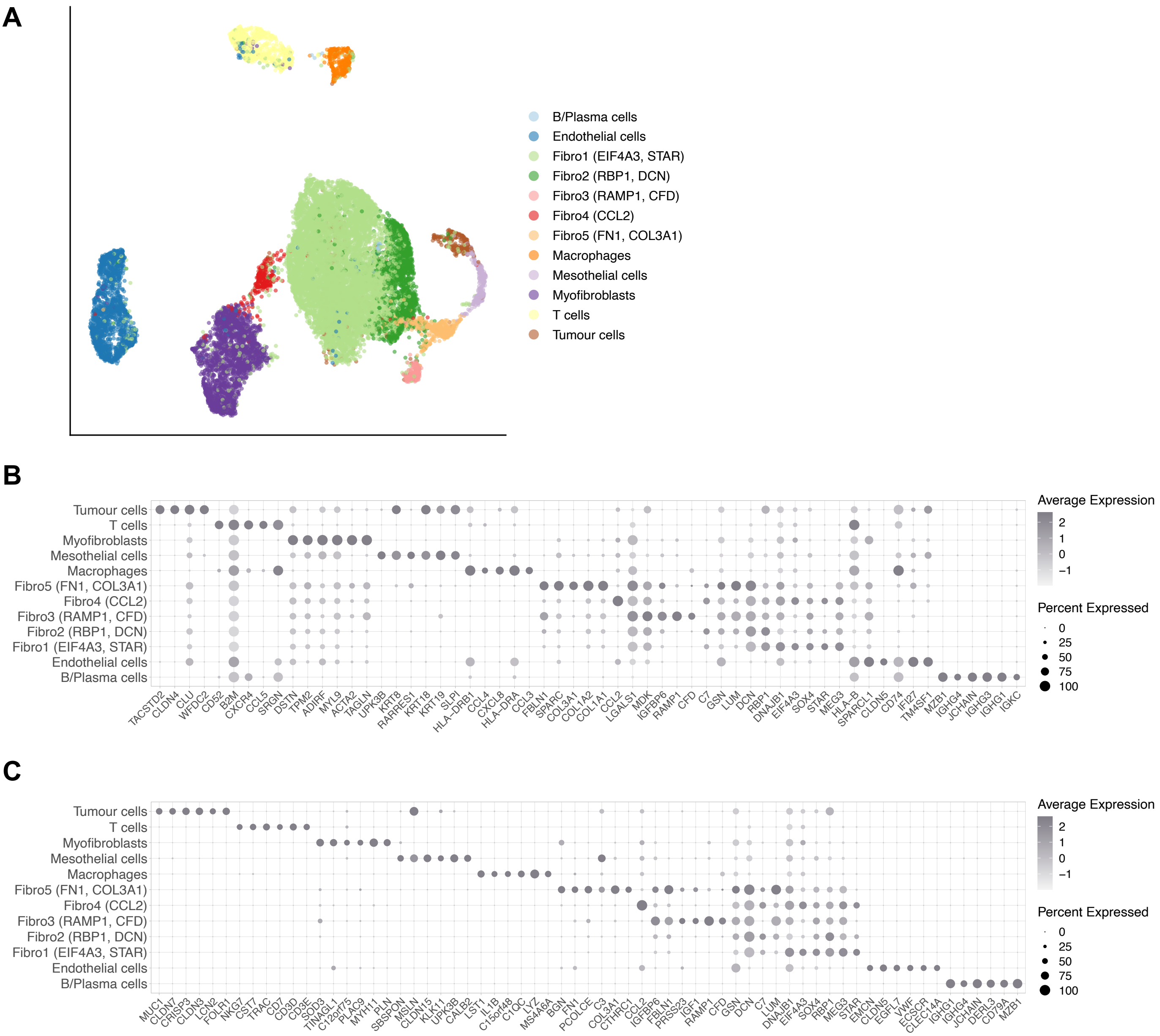

Supplementary Fig. S19: Sankey diagrams summarising the relationship between Visium spot classifications. Gene expression clusters (left) and CNA-based clusters (right) are compared. Shown are also ARI (Adjusted Rand Index) and NMI (Normalised Mutual Information).

Patient 1

ARI 0.39
NMI 0.39

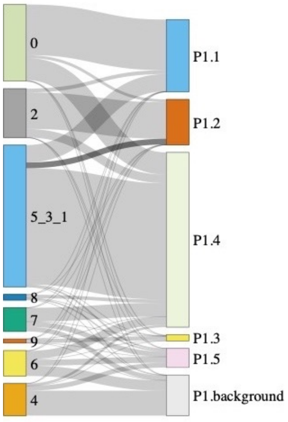

Patient 2

ARI 0.16
NMI 0.26

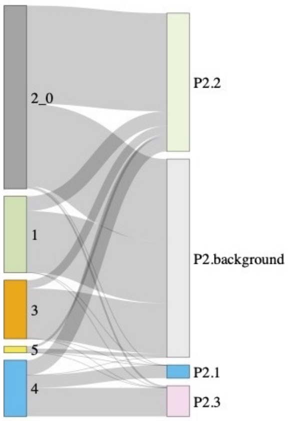

Patient 3

ARI 0.23
NMI 0.31

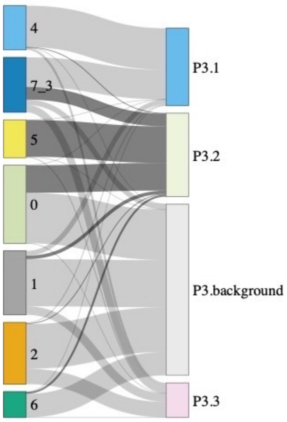

Patient 4

ARI 0.13
NMI 0.23

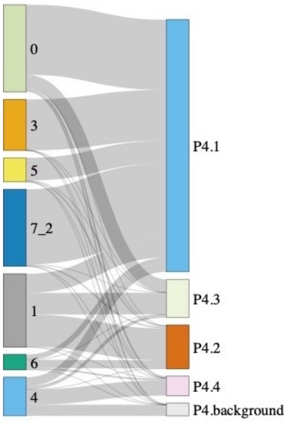

Patient 5

ARI 0.1
NMI 0.19

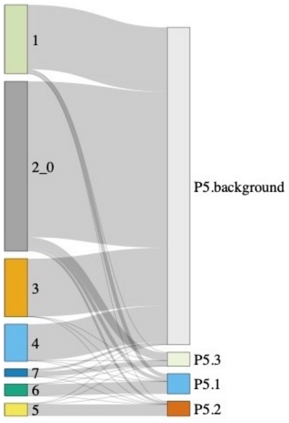

Patient 6

ARI 0.04
NMI 0.12

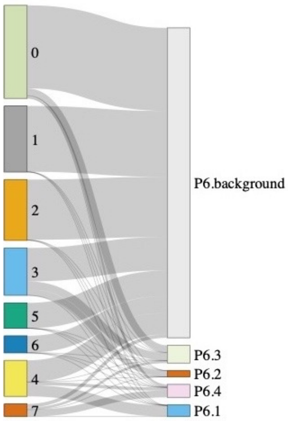

Patient 7

ARI 0.11
NMI 0.15

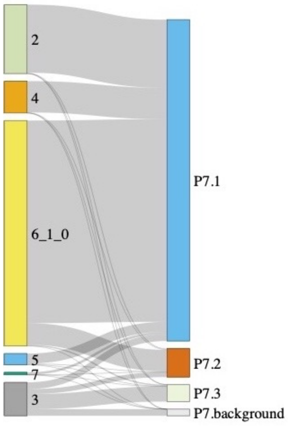

Patient 8

ARI 0.24
NMI 0.29

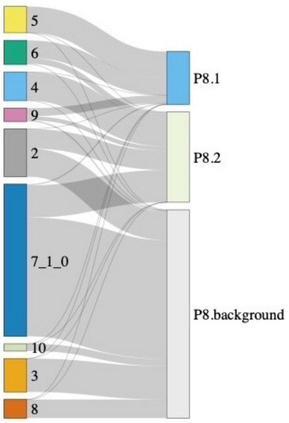

Supplementary Note 1: Use of QuPath histopathological annotations in inferCNV analysis

As an alternative to the use of RCTD to identify background spots lacking tumour cell signal for the inferCNV analyses, we also attempted to use a histopathological approach to classify spots as predominantly overlapping morphologically normal or malignant cells based on the H&E images and QuPath. To do this, we first mapped the location of tumour and non-tumour cells identified by QuPath to spots on the Visium slides (**Supplementary Fig. S20a**). We then ran inferCNV using expression from spots with QuPath tumour spot content of 0% as background and above 50% as tumour. This resulted in substantially weaker and noisier copy number inference. Specifically, there was evidence of CNA signal in the 'normal' spots and loss of signal in the 'tumour spots' (**Supplementary Fig. S20b**).

To assess the impact of background sets we also ran inferCNV using the original background (RCTD tumour weight below 0.15) and spots with QuPath spot tumour percentage above 50%. This substantially improved the inference and predicted the same CNAs as in our original analyses but reduces the number of spots. This suggests that the background spots annotated by QuPath as containing no tumour signal may actually overlap morphologically normal tumour cells. Delving into this further is beyond the scope of the current study.

Supplementary Figure S20: InferCNV analysis of sample from patient 1 using QuPath-based annotation of tumour and normal cells. a) (left) QuPath annotations (red = tumour cells, green = normal), scale bar = 1 mm. **(centre)** % of cells annotated as tumour (red) for each Visium spot, **(right)** Tumour fraction based on RCTD scores. **b**) InferCNV results using QuPath tumour spot content of 0% as background and above 50% as tumour.

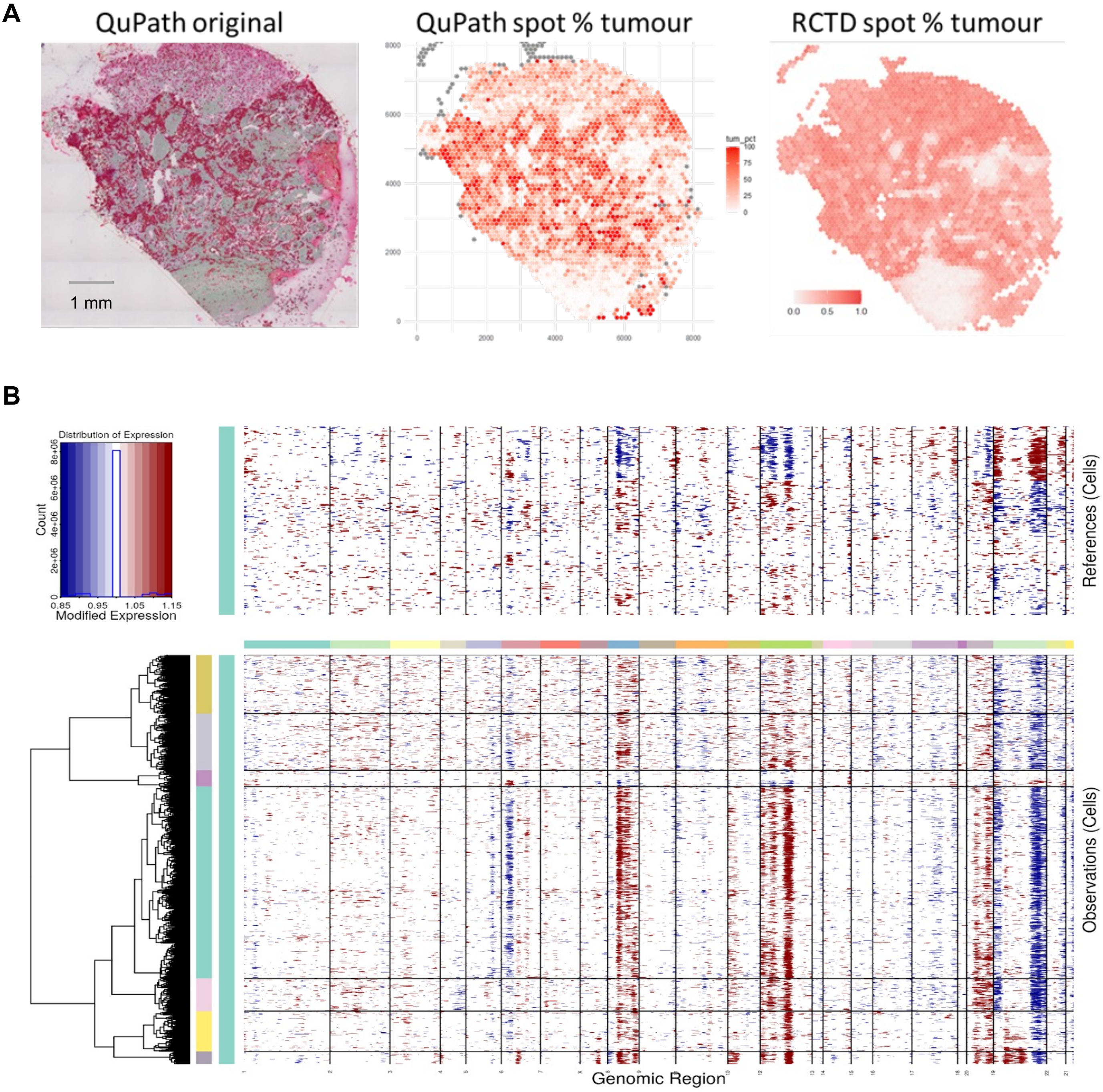

Supplementary Figure S21: InferCNV analysis of sample from patient 1 using QuPath-based annotation of tumour spots and RCTD-based annotation of normal cells. InferCNV results using spots with RCTD tumour weights below 0.15 and QuPath tumour spot content above 50%. Red arrow indicates chr4 deletion seen in subclone P1.2. Blue arrow indicates chr5 deletion seen in subclone P1.1. Yellow arrow indicates chr19 amplification see in subclone P1.3.

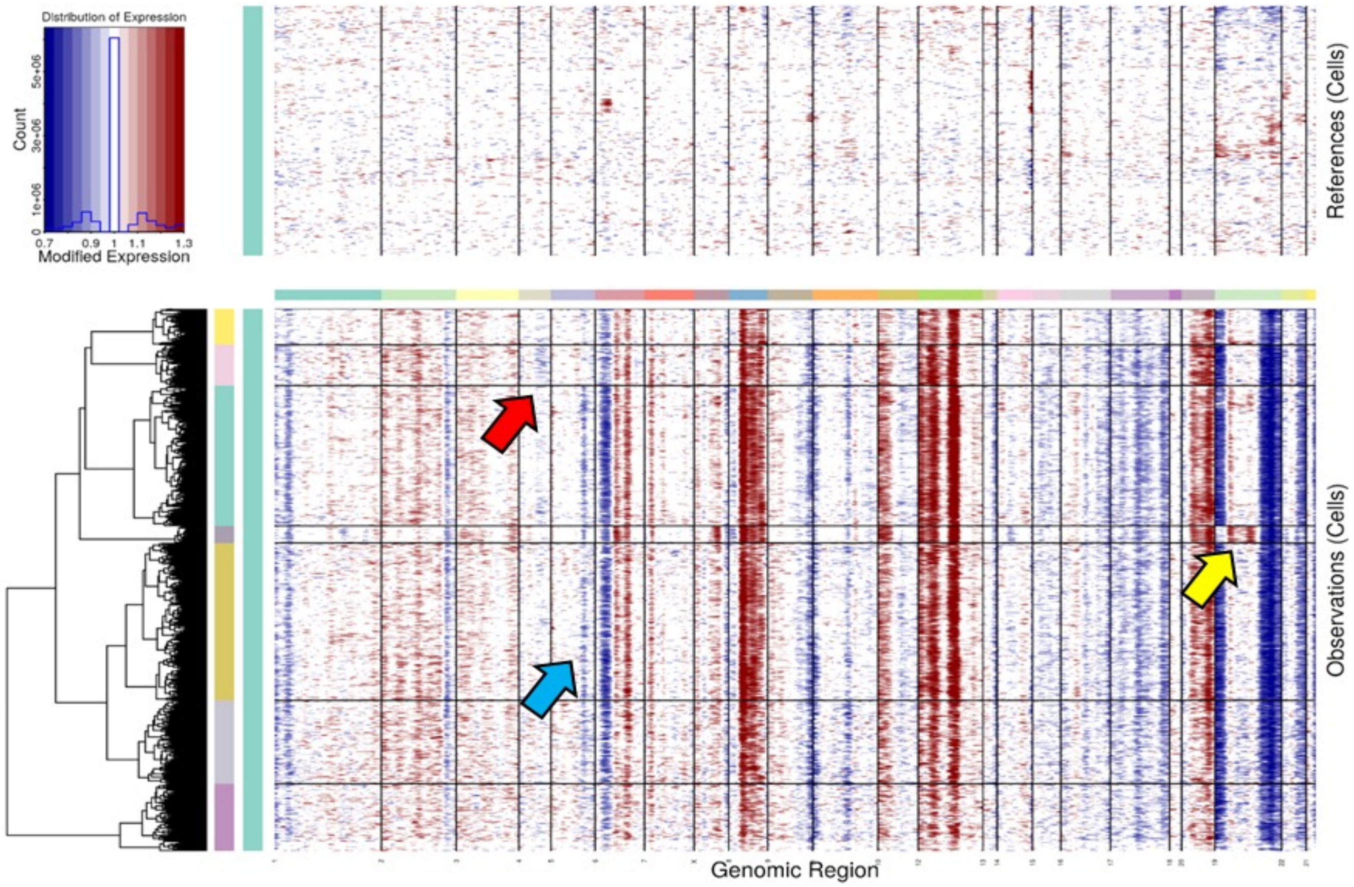

Supplementary Note 2. Chemotherapy response analysis

To investigate possible differences between tumours of good and poor responders, we compared gene expression profiles of Visium spots annotated as malignant based on their CNA profiles in CRS1 (poor responders) and CRS3 (good responders) patients. Differential gene expression analysis showed that in CRS3 (good responders) there were higher expression levels of genes involved in immune response (e.g. immunoglobulins and human leukocyte antigens) and extracellular matrix organisation (e.g. collagens, *DCN, FN1*) (**Supplementary Data 6**). In contrast, genes previously implicated in ovarian cancer progression (*CLU, CD24*)[6-9] and genes encoding ribosomal proteins were overexpressed in CRS1 (poor responder samples) (**Supplementary Data 6**).

We examined the expression of these response-associated genes in our scRNA-seq dataset and found that genes over-expressed in the good responder samples were often specific to B cells and macrophages (**Supplementary Fig. S22a**). In contrast, the genes over-expressed in the poor responder samples were more highly expressed in cancer cells (**Supplementary Fig. S22b**).

RCTD weights observed in malignant spots of CRS1 and CRS3 patients agreed with this, showing higher proportion of tumour cells in the poor responders (**Supplementary Fig. S22c**) and higher proportions of immune cells in the good responders (**Supplementary Fig. S22d**).

Taken together, these results indicate stronger infiltration with immune cells in our good responder samples and higher tumour purity in the poor responder samples.

Supplementary Figure S22: Chemotherapy response analysis. a) Expression in the scRNA-seq dataset of genes associated with good response to chemotherapy, top 20 genes selected based on fold change. **b)** Expression in the scRNA-seq dataset of genes associated with poor response to chemotherapy. **c)** RCTD weights of cancer and non-cancer cells in malignant spots across good (CRS3) and poor (CRS1) responder samples. **d)** Relative RCTD weights of non-cancer cells in malignant spots across good (CRS3) and poor (CRS1) responder samples. In (c) and (d), on each box, the central line indicates the median, the lower and upper bounds correspond to the first and third quartiles (the 25th and 75th percentiles), the whiskers extend to the values no further than 1.5 * IQR from the box bounds (where IQR is the interquartile range, or distance between the first and third quartiles), data beyond the end of the whiskers (outliers) are plotted individually.

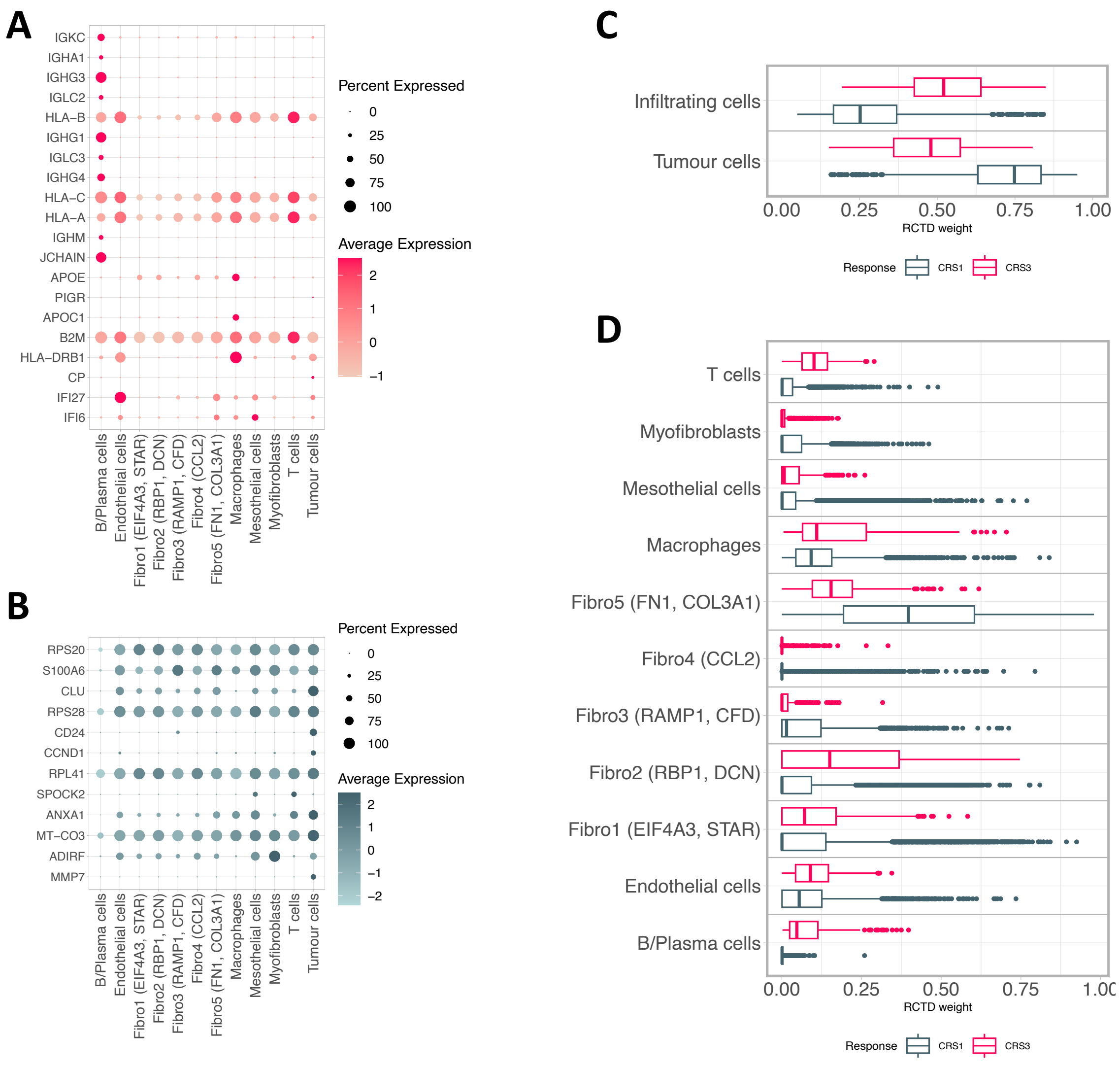

Supplementary Note 3. Cell-to-cell communication underlying different infiltration patterns

Notably, 41 of the 249 genes differentially expressed between malignant clusters (with FDR < 0.05, $|\log_2FC| > 1$) encoded ligands and 15 encoded receptors (significant overrepresentation with p-values of $2.3*10^{-15}$ and 0.01, respectively, hypergeometric test). Given this enrichment, we next sought to examine whether tumour-cell-derived ligands may be associated with infiltration by non-malignant cell types. To address this, we first extracted 73 robust tumour-cell-derived ligands that were detected in all Visium samples and most highly expressed in tumour cells according to both our and a larger recently published metastatic ovarian cancer scRNA-seq dataset[1] (**Supplementary Fig. S23**).

We next calculated correlations between the expression levels of these 73 ligands and adjusted RCTD weights (normalised to sum up to 1 for non-tumour cell types) for each non-tumour cell type across all spots in the dataset (**Supplementary Data 10**). The expression of all 73 ligands was significantly correlated (FDR ≤ 0.05) with the adjusted RCTD weights of at least one non-tumour cell type. Focusing on the top 43 ligand-cell type pairs with significant correlation coefficients ≤ -0.2 or ≥ 0.2, involving 18 ligands, revealed several were strongly correlated with multiple cell types. For example, *CXCL10* was positively correlated with adjusted RCTD weights for B/plasma cells, macrophages and T cells, while *LTBP3* was correlated with a fibroblast population and anti-correlated with macrophages (**Supplementary Data 10**). Interestingly, *CD24*, *LCN2,* and *SLPI* shared significant correlations with a common set of cells (positive correlations with multiple fibroblast populations and mesothelial cells and anti-correlation with B/plasma cells). Notably, 14 of these 18 ligands were differentially expressed between malignant clones of at least one patient.

In order to explore the possibility that the connections we observed between tumour-derived ligands and adjusted RCTD weights of a specific cell type may be a result of direct communication through a receptor expressed by the infiltrating cell population, we employed connectomeDB2020[10] to retrieve the corresponding receptors for each ligand and check their expression in the single cell dataset. For 9 of the 43 most highly correlated ligand-cell type pairs (correlations ≤ -0.2 or ≥ 0.2, FDR ≤ 0.05), a cognate receptor was detected in at least 10% of the cells in the correlated cell type and, thus, represents a potential direct signalling path (**Supplementary Data 10**).

One potential direct signalling path identified was *CXCL10* signalling to T cells and B/plasma cells expressing the cognate receptor *CXCR3* (also confirmed in the independent dataset from Zhang *et al.*[1], **Supplementary Fig. S24**). This is consistent with *CXCL10's* role as a chemoattractant and a report that its expression correlates with tumour infiltrating lymphocytes (TILs) and doubled overall survival in HGSOC[11]. Notably, *CXCL10* was differentially expressed between malignant clusters P5.1 and P5.2 of patient 5 which raised the possibility that T and B cell infiltration patterns may differ between the tumour subclones predicted within. Indeed, B/plasma cell infiltration was significantly different between P5.1 and P5.2, based on the permutation testing of RCTD cell type weights, however, no significance was observed for T cells (**Supplementary Fig. S14**).

Supplementary Figure S23: Expression and detection rates of 73 tumour-cell-derived ligands in our (a) and Zhang *et al.*[1] (b) scRNA-seq datasets. EOC, epithelial ovarian carcinoma.

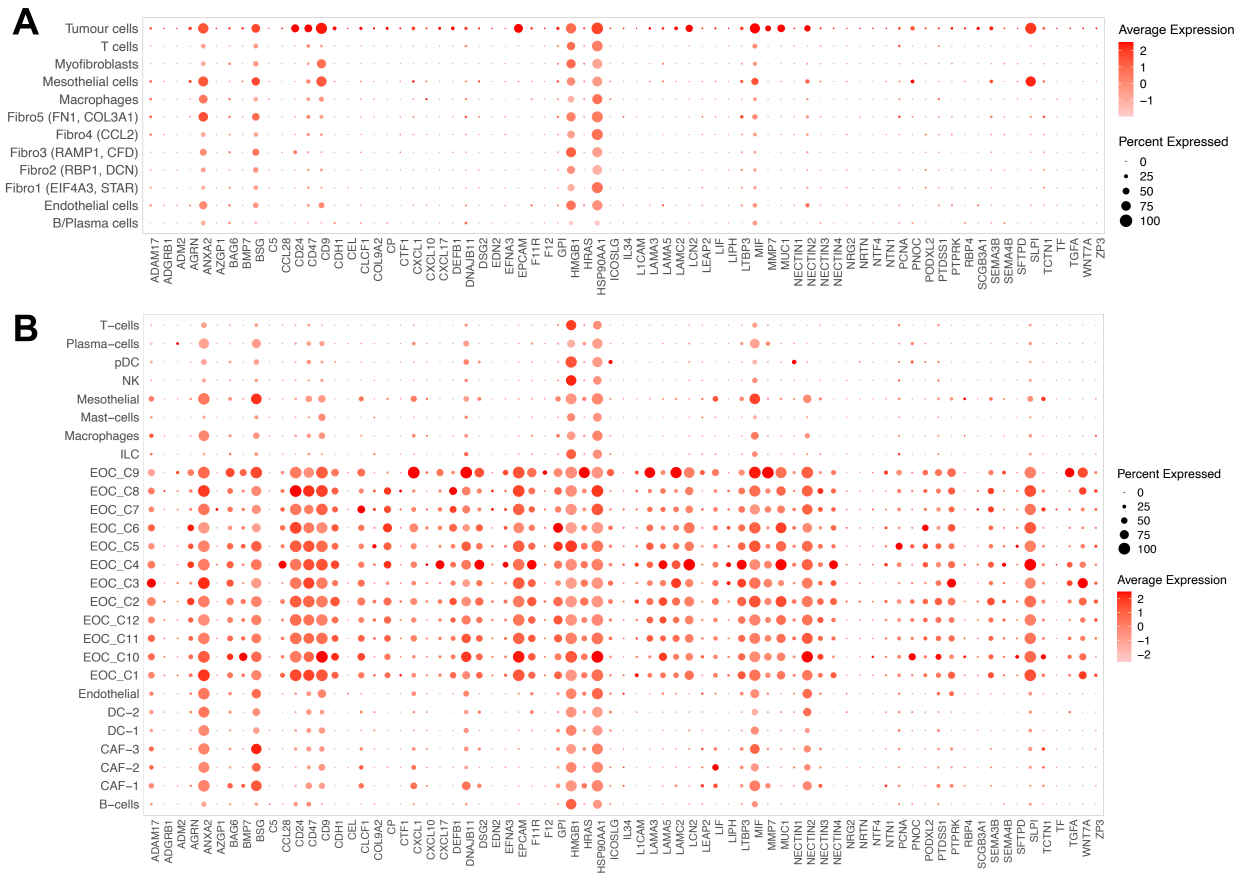

Supplementary Figure S24: Expression and detection rates of *CXCL10-CXCR3* ligand-receptor pair in Zhang *et al.*[1] scRNA-seq dataset.

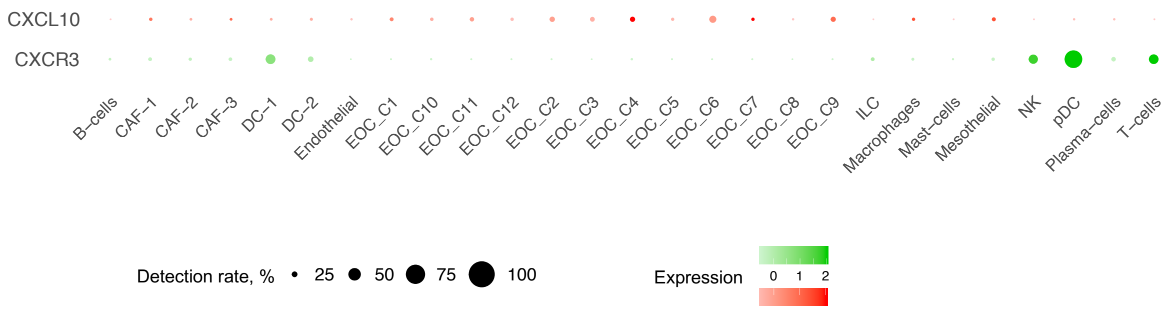

Supplementary References

1. Zhang, K. et al. Longitudinal single-cell RNA-seq analysis reveals stress-promoted chemoresistance in metastatic ovarian cancer. Sci Adv 8, eabm1831 (2022).
2. Gao, J. et al. Integrative analysis of complex cancer genomics and clinical profiles using the cBioPortal. Sci. Signal. 6, l1 (2013).
3. Stur, E. et al. Spatially resolved transcriptomics of high-grade serous ovarian carcinoma. iScience 25, 103923 (2022).
4. Ferri-Borgogno, S. et al. Spatial Transcriptomics Depict Ligand-Receptor Cross-talk Heterogeneity at the Tumor-Stroma Interface in Long-Term Ovarian Cancer Survivors. Cancer Res. 83, 1503–1516 (2023).
5. Olbrecht, S. et al. High-grade serous tubo-ovarian cancer refined with single-cell RNA sequencing: specific cell subtypes influence survival and determine molecular subtype classification. Genome Med. 13, 111 (2021).
6. Tarhriz, V. et al. Overview of CD24 as a new molecular marker in ovarian cancer. J. Cell. Physiol. 234, 2134–2142 (2019).
7. Hassan, M. K. et al. Clusterin is a potential molecular predictor for ovarian cancer patient’s survival: targeting clusterin improves response to paclitaxel. J. Exp. Clin. Cancer Res. 30, 113 (2011).
8. Wei, L. et al. Roles of clusterin in progression, chemoresistance and metastasis of human ovarian cancer. Int. J. Cancer 125, 791–806 (2009).
9. Fu, Y. et al. Overexpression of clusterin promotes angiogenesis via the vascular endothelial growth factor in primary ovarian cancer. Mol. Med. Rep. 7, 1726–1732 (2013).
10. Hou, R., Denisenko, E., Ong, H. T., Ramilowski, J. A. & Forrest, A. R. R. Predicting cell-to-cell communication networks using NATMI. Nat. Commun. 11, 5011 (2020).
11. Bronger, H. et al. CXCL9 and CXCL10 predict survival and are regulated by cyclooxygenase inhibition in advanced serous ovarian cancer. Br. J. Cancer 115, 553–563 (2016).
