# Supplementary material for: Spatial transcriptomics reveals discrete tumour microenvironments and autocrine loops within ovarian cancer subclones
Source: Nat Commun. 2024 Apr 3;15:2860. doi: 10.1038/s41467-024-47271-y (PMC10991508; doi:10.1038/s41467-024-47271-y)
Supplement: Supplementary file 3 — Description of Additional Supplementary Files [file 41467_2024_47271_MOESM3_ESM.pdf]

Description of Additional Supplementary Files

Supplementary Data 1: Sample information for Visium and scRNA-seq samples.

Supplementary Data 2: High-confidence CNAs identified in each CNA-based cluster using inferCNV.

Supplementary Data 3: IchorCNA results for regions corresponding to the clusters P1.1, P1.2, P1.3, and P1.background.

Supplementary Data 4: Differentially expressed genes (DEGs) calculated between pairs of CNA-based clusters containing putative malignant subclones for each patient. Differential expression (DE) testing was performed using two-sided non-parametric Wilcoxon rank sum test in Seurat. P-values were adjusted based on Bonferroni correction using all features in the dataset. Seurat *FindMarkers* function was used with logfc.threshold = 0.5, min.pct = 0.5 and otherwise default parameters. Adjusted p-value threshold of 0.05 was used.

Supplementary Data 5: For each within-patient pairwise comparison of malignant clusters, the table shows DEGs identified as over-expressed and located in the amplified high-confidence CNAs reported for the corresponding cluster.

Supplementary Data 6: Genes differentially expressed between malignant spots of Visium samples of CRS1 and CRS3 patients. Differential expression (DE) testing was performed using a two-sided nonparametric Wilcoxon rank sum test in Seurat. P-values were adjusted based on Bonferroni correction using all features in the dataset.

Supplementary Data 7: Genes differentially expressed between *PIGR*+ tumour cells and *PTGS1*+ tumour cells in the CosMx SMI dataset.

Supplementary Data 8: Genes present on both Visium and CosMx platforms and significantly differentially expressed between tumour subclones on at least one of these platforms.

Supplementary Data 9: Neighbourhood analysis of cell types in the CosMx data. Matrices summarise the proportions of each cell type neighbouring each other cell type at 3 different distances (radii of 110, 340, and 648 pixels) which yielded median numbers of 3, 30 and 100 neighbouring cells. Neighbouring cells were surveyed using Squidpy[45]. Monte Carlo procedure was used to calculate empirical P values which were then corrected for multiple testing using Benjamini-Hochberg procedure.

Supplementary Data 10: Correlation between 73 robust tumour-cell-derived ligands and adjusted RCTD weights for each non-tumour cell type, calculated across all spots in the Visium dataset. Includes the subset of top 43 ligand-cell type pairs as a separate tab.

Supplementary Data 11: Cell type labels assigned to individual cells in the scRNA-seq dataset.
