# Supplementary material for: Spatial transcriptomics reveals discrete tumour microenvironments and autocrine loops within ovarian cancer subclones
Source: Nat Commun. 2024 Apr 3;15:2860. doi: 10.1038/s41467-024-47271-y (PMC10991508; doi:10.1038/s41467-024-47271-y)
Supplement: Supplementary file 15 — Reporting Summary [file 41467_2024_47271_MOESM15_ESM.pdf]

nature portfolio

Corresponding author(s): Elena Denisenko, Paul A. Cohen, Yu Yu, Alistair R. R. Forrest

Last updated by author(s): Jan 5, 2024

Reporting Summary

Nature Portfolio wishes to improve the reproducibility of the work that we publish. This form provides structure for consistency and transparency in reporting. For further information on Nature Portfolio policies, see our Editorial Policies and the Editorial Policy Checklist.

Statistics

For all statistical analyses, confirm that the following items are present in the figure legend, table legend, main text, or Methods section.

n/a	Confirmed	
☐	☒	The exact sample size (*n*) for each experimental group/condition, given as a discrete number and unit of measurement
☐	☒	A statement on whether measurements were taken from distinct samples or whether the same sample was measured repeatedly
☐	☒	The statistical test(s) used AND whether they are one- or two-sided<br>*Only common tests should be described solely by name; describe more complex techniques in the Methods section.*
☒	☐	A description of all covariates tested
☒	☐	A description of any assumptions or corrections, such as tests of normality and adjustment for multiple comparisons
☒	☐	A full description of the statistical parameters including central tendency (e.g. means) or other basic estimates (e.g. regression coefficient) AND variation (e.g. standard deviation) or associated estimates of uncertainty (e.g. confidence intervals)
☐	☒	For null hypothesis testing, the test statistic (e.g. *F*, *t*, *r*) with confidence intervals, effect sizes, degrees of freedom and *P* value noted<br>*Give P values as exact values whenever suitable.*
☒	☐	For Bayesian analysis, information on the choice of priors and Markov chain Monte Carlo settings
☒	☐	For hierarchical and complex designs, identification of the appropriate level for tests and full reporting of outcomes
☒	☐	Estimates of effect sizes (e.g. Cohen's *d*, Pearson's *r*), indicating how they were calculated

Our web collection on statistics for biologists contains articles on many of the points above.

Software and code

Policy information about availability of computer code

Data collection	Cell Ranger 3.0.2, Space Ranger 1.0.0
Data analysis	Most software used in this manuscript are publicly available and fully described in the methods section of the manuscript. The NanoString CosMx data and custom code are available from Zenodo (https://zenodo.org/records/10048057).

For manuscripts utilizing custom algorithms or software that are central to the research but not yet described in published literature, software must be made available to editors and reviewers. We strongly encourage code deposition in a community repository (e.g. GitHub). See the Nature Portfolio guidelines for submitting code & software for further information.

Data

Policy information about availability of data

All manuscripts must include a data availability statement. This statement should provide the following information, where applicable:

- Accession codes, unique identifiers, or web links for publicly available datasets
- A description of any restrictions on data availability
- For clinical datasets or third party data, please ensure that the statement adheres to our policy

The scRNA-seq and Visium data are available from the Gene Expression Omnibus (GEO) repository with the primary accession code https://www.ncbi.nlm.nih.gov/ geo/query/acc.cgi?acc=GSE211956 (GSE211956). The NanoString CosMx data and code are available from Zenodo (https://zenodo.org/records/10048057). Source data for plots in Figure 3 are provided with this paper. The low pass whole genome sequence (WGS) data is deposited at dbGaP under accession code phs003561.v1.p1 (https://www.ncbi.nlm.nih.gov/projects/gap/cgi-bin/study.cgi?study_id=phs003561.v1.p1). The WGS data is available under restricted access to protect the donor's privacy. Access to the data for cancer research purposes is via dbGaP and can be requested by permanent employees of an institution at a level equivalent to a tenure-track professor or senior scientist with laboratory administration and oversight responsibilities. The requests are managed by the Data Access Committee of the NCI, and after approval, access is granted for 12 months.

The two scRNA-seq publicly available datasets used in this study are available in the Gene Expression Omnibus (GEO) database under accession code GSE165897 (https://www.ncbi.nlm.nih.gov/geo/query/acc.cgi?acc=GSE165897) [Zhang et al. (https://doi.org/10.1158/10.1126/sciadv.abm183)] AND from http://blueprint.lambrechtslab.org [Olbrecht et al. (https://doi.org/10.1158/10.1186/s13073-021-00922-x)].

The two spatial transcriptomics publicly available datasets used in this study are available in the Gene Expression Omnibus (GEO) database under accession code GSE189843 (https://www.ncbi.nlm.nih.gov/geo/query/acc.cgi?acc=GSE189843) [Stur et al. (https://doi.org/10.1016/j.isci.2022.103923)] AND from CodeOcean (https://codeocean.com/capsule/1912679/tree/v1) [Ferri-Borgogno et al. (https://doi.org/10.1158/0008-5472.CAN-22-1821)].

Research involving human participants, their data, or biological material

Policy information about studies with human participants or human data. See also policy information about sex, gender (identity/presentation), and sexual orientation and race, ethnicity and racism.

Reporting on sex and gender	This study was on ovarian cancer from female participants only.
Reporting on race, ethnicity, or other socially relevant groupings	N/A
Population characteristics	High-grade serous ovarian tumours from eight patients diagnosed with stage III-IV cancers were included in this study. Patients were treated with 3-6 cycles of platinum-based chemotherapy. All tumour samples were derived from ovarian sites during interval debulking surgery.
Recruitment	Participants were recruited in a clinical setting prior to surgery based on the population characteristics described above. All participants were given information about the study and provided written informed consent before enrolment. We are unaware of any potential self selection biases.
Ethics oversight	The study was approved by St John of God Health Care (SJGHC), The University of Western Australia (UWA) and Curtin University Human Research Ethics Committees (#1217 and RA/4/20/5784).

Note that full information on the approval of the study protocol must also be provided in the manuscript.

Field-specific reporting

Please select the one below that is the best fit for your research. If you are not sure, read the appropriate sections before making your selection.

☒ Life sciences ☐ Behavioural & social sciences ☐ Ecological, evolutionary & environmental sciences

For a reference copy of the document with all sections, see nature.com/documents/nr-reporting-summary-flat.pdf

Life sciences study design

All studies must disclose on these points even when the disclosure is negative.

Sample size	No sample size calculation was performed. 8 samples were profiled due to cost and sample availability.
Data exclusions	For the scRNA-seq: Cells with at least 500 genes, at least 1000 UMIs and under 15% of mitochondrial reads were retained. Also genes detected in less than 3 cells were discarded.<br>For the Visium spatial transcriptomics: barcodes with less than 400 genes were excluded.
Replication	The observation that multiple CNA subclones are observed in HGSOC sections was replicated across 5 of 8 donors from this study, and in 2 of 4 donors from the Ferri-Borgogno et al. dataset.<br><br>There was no replication of Visium data for each donor.<br><br>For donor 1 low pass whole genome sequencing was carried out using 2 to 3 replicates for each subclone.<br><br>For the CosMx data there were no replicates.<br><br>Note different patients have different CNAs and subclones. We also validated this in one patient by low pass WGS.
Randomization	This was not relevant to this study. 8 samples were spatially profiled, there were no test and control groups.
Blinding	This was not relevant to this study. There are no groups.

Reporting for specific materials, systems and methods

We require information from authors about some types of materials, experimental systems and methods used in many studies. Here, indicate whether each material, system or method listed is relevant to your study. If you are not sure if a list item applies to your research, read the appropriate section before selecting a response.

Materials & experimental systems

n/a	Involved in the study
☐	☒ Antibodies
☒	☐ Eukaryotic cell lines
☒	☐ Palaeontology and archaeology
☒	☐ Animals and other organisms
☐	☒ Clinical data
☒	☐ Dual use research of concern
☒	☐ Plants

Methods

n/a	Involved in the study
☒	☐ ChIP-seq
☒	☐ Flow cytometry
☒	☐ MRI-based neuroimaging

Antibodies

Antibodies used	A 4-fluorophore-conjugated antibody cocktail from Nanostring against CD298/B2M (488 nm), PanCK (532 nm), CD45 (594 nm), and CD3 (647 nm) proteins was used in the CosMx SMI instrument.
Validation	The antibody cocktail was validated by Nanostring for use in the CosMx.

Clinical data

Policy information about clinical studies
All manuscripts should comply with the ICMJE guidelines for publication of clinical research and a completed CONSORT checklist must be included with all submissions.

Clinical trial registration	N/A - Clinical samples were provided but not part of a clinical trial
Study protocol	N/A
Data collection	N/A
Outcomes	N/A - Clinical samples were provided but not part of a clinical trial

Plants

Seed stocks	*Report on the source of all seed stocks or other plant material used. If applicable, state the seed stock centre and catalogue number. If plant specimens were collected from the field, describe the collection location, date and sampling procedures.*
Novel plant genotypes	*Describe the methods by which all novel plant genotypes were produced. This includes those generated by transgenic approaches, gene editing, chemical/radiation-based mutagenesis and hybridization. For transgenic lines, describe the transformation method, the number of independent lines analyzed and the generation upon which experiments were performed. For gene-edited lines, describe the editor used, the endogenous sequence targeted for editing, the targeting guide RNA sequence (if applicable) and how the editor was applied.*
Authentication	*Describe any authentication procedures for each seed stock used or novel genotype generated. Describe any experiments used to assess the effect of a mutation and, where applicable, how potential secondary effects (e.g. second site T-DNA insertions, mosiacism, off-target gene editing) were examined.*
